# Full-Length Transcriptional Analysis of the Same Soybean Genotype With Compatible and Incompatible Reactions to *Heterodera glycines* Reveals Nematode Infection Activating Plant Defense Response

**DOI:** 10.3389/fpls.2022.866322

**Published:** 2022-05-18

**Authors:** Minghui Huang, Ye Jiang, Ruifeng Qin, Dan Jiang, Doudou Chang, Zhongyan Tian, Chunjie Li, Congli Wang

**Affiliations:** ^1^Key Laboratory of Soybean Molecular Design Breeding, Northeast Institute of Geography and Agroecology, Chinese Academy of Sciences, Harbin, China; ^2^Heilongjiang Academy of Agricultural Sciences, Daqing, China

**Keywords:** full-length transcriptome sequencing, *glycine max*, *Heterodera glycines*, incompatible and compatible response, VQ–WRKY interaction

## Abstract

Full-length transcriptome sequencing with long reads is a powerful tool to analyze transcriptional and post-transcriptional events; however, it has not been applied on soybean (*Glycine max*). Here, a comparative full-length transcriptome analysis was performed on soybean genotype 09-138 infected with soybean cyst nematode (SCN, *Heterodera glycines*) race 4 (SCN4, incompatible reaction) and race 5 (SCN5, compatible reaction) using Oxford Nanopore Technology. Each of 9 full-length samples collected 8 days post inoculation with/without nematodes generated an average of 6.1 GB of clean data and a total of 65,038 transcript sequences. After redundant transcripts were removed, 1,117 novel genes and 41,096 novel transcripts were identified. By analyzing the sequence structure of the novel transcripts, a total of 28,759 complete open reading frame (ORF) sequences, 5,337 transcription factors, 288 long non-coding RNAs, and 40,090 novel transcripts with function annotation were predicted. Gene Ontology (GO) and Kyoto Encyclopedia of Genes and Genomes (KEGG) enrichment analyses of differentially expressed genes (DEGs) revealed that growth hormone, auxin-activated signaling pathway and multidimensional cell growth, and phenylpropanoid biosynthesis pathway were enriched by infection with both nematode races. More DEGs associated with stress response elements, plant-hormone signaling transduction pathway, and plant–pathogen interaction pathway with more upregulation were found in the incompatible reaction with SCN4 infection, and more DEGs with more upregulation involved in cell wall modification and carbohydrate bioprocess were detected in the compatible reaction with SCN5 infection when compared with each other. Among them, overlapping DEGs with a quantitative difference was triggered. The combination of protein–protein interaction with DEGs for the first time indicated that nematode infection activated the interactions between transcription factor WRKY and VQ (valine-glutamine motif) to contribute to soybean defense. The knowledge of the SCN–soybean interaction mechanism as a model will present more understanding of other plant–nematode interactions.

## Introduction

Soybean cyst nematode (SCN, *Heterodera glycines* Ichinohe) is one of the most economically important diseases in soybean (*Glycine max* L. Merrill) worldwide. The latest statistical analysis of estimated cumulative soybean economic losses due to diseases in 28 states within the United States from 1996 to 2016 indicated that SCN accounted for 23.2% of the total losses (US$ 73,535 per hectare) which was top-ranked, far greater than the second disease charcoal rot (10%) (Bandara et al., 2020). In China, annual economic losses in soybean could reach up to US$ 120 million (Li Y. et al., 2011). SCN is a soil-borne sedentary parasitic nematode in the major host soybean. In the only free-moving stage of SCN, a second-stage juvenile (J2) hatches from an egg (first-stage juvenile inside the egg) to search for host roots through signals released from plant roots, moves to root tips, and penetrates roots using a stylet. The J2 moves inside the root, establishes a feeding site, and reprograms host root cells by directing gland secretions into plants that can dissolve cell walls and fuse the protoplast of neighboring cells, and eventually forms a unique feeding structure called syncytium as a nutrient source for nematode development, consequently suppressing plant growth and affecting yield (Niblack et al., 2006; Mitchum and Baum, 2008).

Host-plant resistance combined with crop rotation is the most effective way to control SCN. More than 300 quantitative trait loci (QTLs) associated with SCN resistance were mapped to 20 chromosomes (chr) (soybase.org), but only two major resistance genes, *rhg1* on chromosome (chr) 18 and *Rhg4* on chr 8, were cloned and characterized (Cook et al., 2012, 2014; Liu et al., 2012, 2017). The presence of multiple minor QTLs contributing to resistance in either resistant or susceptible soybean plants makes resistance breeding more difficult than expected (Huang et al., 2021). Furthermore, shifts in virulence have caused a decrease or loss of resistance because of the long-term planting of a single source of resistance varieties, e.g., 90% of resistance sources derived from the PI88788 background in the United States and mainly Peking resistance sources in China (Mitchum et al., 2007; Niblack et al., 2008; Acharya et al., 2016; Hua et al., 2018; Huang et al., 2022). Furthermore, Peking and PI88788 display resistance only to some SCN races or HG Types. The lack of broad resistance sources and the presence of multiple SCN races or HG types in the field result in SCN spreading widely and quickly. Thus, understanding the molecular mechanisms of SCN infection and plant resistance will gain more insights to develop new control strategies, including engineering important candidate genes to increase resistance.

Plants have evolved to develop two layers of pathogen defense immune systems: the first layer is pathogen- or microbe-associated molecular pattern (PAMP/MAMP) triggered immunity (PTI); the second layer is effector-triggered immunity (ETI), which fits species-specific disease resistance (Dangl and Jones, 2001; Eitas and Dangl, 2010; Monaghan and Zipfel, 2012). The plant cell wall surface contains pattern recognition receptors (PRRs) that can detect pathogen or microbe structures to activate PTI, and phytohormones as defense-related signaling molecules, such as salicylic acid (SA), jasmonate acid (JA), ethylene (ET), induce plant to produce pathogenesis-related (PR) proteins, e.g., β-1,3-glucanases, peroxidase, oxidase-like, thionin, and proteinase inhibitor, etc. (Sels et al., 2008). ETI is initiated by intracellular nucleotide-binding protein domain leucine-rich repeat proteins (NLRs) to generate a hypersensitive response (HR) with local cell death (Eitas and Dangl, 2010). PTI and ETI are activated by two distinct classes of receptors in early signaling, but recent evidence suggests that PTI and ETI can crosstalk in downstream response, although how they contribute to immunity with quantitative and/or qualitative outputs is still undefined (Naveed et al., 2020; Yuan et al., 2021). Transcriptome analysis of different pathosystems denotes that both compatible and incompatible interactions are able to trigger an overlapping change of gene expression but with quantitative differences (Mine et al., 2018; Yuan et al., 2021).

Studies have been performed on compatible and incompatible interactions between SCN and soybean roots based on microarray and RNA-seq transcriptome analyses (Ithal et al., 2007; Klink et al., 2007; Puthoff et al., 2007; Klink and Matthews, 2009; Kandoth et al., 2011; Li X. et al., 2011; Li et al., 2012, 2018; Mazarei et al., 2011; Wan et al., 2015; Zhang et al., 2017; Kang et al., 2018; Neupane et al., 2019; Song et al., 2019; Jiang et al., 2020; Miraeiz et al., 2020). All sequence annotations revealed that SCN infection can induce or suppress gene expression either in susceptible or resistant cultivars, and a series of defense genes (*PPRs* and *NLRs*), *MAPK* (mitogen-activated protein kinase) signaling cascade, *WRKY* and *MYB* transcription factors (TFs), heat shock protein (*HSP*) genes, *PR* genes, and phenylpropanoid metabolism genes have been identified but with variance depending on SCN race/HG type and plant type.

RNA-sequencing (RNA-seq) based on high-throughput next-generation sequencing (NGS) (e.g., Illumina) has been used widely to measure differential gene expression because it is a cost-effective and advanced technology (Finotello and Di Camillo, 2015). However, RNA-seq requires fragmentation of RNA or cDNA to generate short reads when preparing samples, which diminishes the information from original full-length transcripts; thus, it is harder to obtain post/co-transcriptional processing events that are responsible for producing a mature RNA molecule that can leave the nucleus to function in the cell by chemical structure alteration of the RNA primary transcript (Kiss, 2001). Advanced full-length transcriptome sequencing with longer reads circumvents these challenges. Currently, PacBio and Oxford Nanopore (ONT) are the most popular 3rd-generation full-length sequencing technologies that can provide more complex transcription and reveal the real structure of sequences during transcription, such as alternative splicing (AS), alternative polyadenylation (APA), and long non-coding RNA (lncRNA) and gene fusion, which can increase the complexity of the transcriptome and proteome. Compared with PacBio sequencing, ONT uses ion current blockades to directly sequence more long-native DNA or full-length RNA molecules (Cui et al., 2020; Xie et al., 2021). AS, one of the important steps in post-transcriptional modification, can recognize and eliminate intronic regions of a precursor messenger RNA (pre-mRNA) to generate multiple mRNAs to regulate gene expression that consequently promotes proteome diversity. AS plays key roles not only in plant growth and development but also in response to biotic or abiotic stimuli or adaptation (Matsukura et al., 2010; Severing et al., 2012; Syed et al., 2012; Mandadi and Scholthof, 2015; Wang et al., 2018; Bedre et al., 2019; Martín et al., 2021). APA can produce multiple mRNA polyadenylation isoforms through pre-mRNA endonucleolytic cleavage and poly(A) tail addition at the 3’ cleavage site end of a nascent transcript to change the length of untranslated regions (UTRs) or coding regions that may influence mRNA stability, translation efficiency, subcellular localization, or gene function gain or loss. Consequently, all these changes will result in various plant physiological and biochemical processes (Yeh and Yong, 2016; Sadek et al., 2019; Zhang et al., 2020; Tu et al., 2021); for example, APA participates in cell wall modification, root hair development, DNA repair, and gene regulation in response to abiotic and biotic stresses (Cao et al., 2019; Ye et al., 2019; Yan et al., 2021). LncRNAs longer than 200 nucleotides are epigenetic regulators that regulate gene expression by interacting with mRNAs, DNAs, proteins, and miRNAs to participate in biological processes such as plant growth and development and biotic and abiotic stress responses (Budak et al., 2020; Yu et al., 2020; Tu et al., 2021; Urquiaga et al., 2021).

The application of the full-length transcriptome sequencing technique in plants is limited compared with second-generation sequencing because of its higher cost. Currently, full-length transcriptome sequencing for inferring and improving gene models and identifying novel genes has been reported on rice, wheat, maize, cotton, pecan, poplar, and others but not on soybean (Clavijo et al., 2017; Wang et al., 2018; Zhang et al., 2019; Zhao et al., 2019; Li C. et al., 2020; Yang et al., 2021).

Our previous study has demonstrated that the soybean breeding line 09-138, developed in northeast China, carries the *rhg1-a* and *Rhg4-b* loci and has resistance to SCN race 4 (SCN4, HG type 1.2.3.5.6.7) but has susceptibility to SCN race 5 (SCN5, HG type 2.5.7), which is different from the Peking (*rhg1-a* + *Rhg4-a*) and PI88788 (*rhg1-b* + *Rhg4-b*) resistance backgrounds (Hua et al., 2018; Huang et al., 2022). We postulated that differences in response to the two SCN races should be related to distinct transcriptional responses in the early stages of nematode infection as described above. To test this, line 09-138 was inoculated with the two SCN races, and nematode development was observed inside the roots. Comparative full-length transcriptome analysis of soybean breeding lines 09-138 infected with SCN4 (resistance response) and SCN5 (susceptibility response) was performed using the ONT technology. We investigated AS and APA events and identified lncRNA and transcription factors in detected novel transcripts. Then, differentially expressed genes (DEGs) and transcripts (DETs) were analyzed, and DEGs associated with stress response elements were explored. Enriched DEG-GO (Gene Ontology) terms and DEG-KEGG (Kyoto Encyclopedia of Genes and Genomes) pathways were compared between resistant and susceptible responses. Protein–protein interactions were predicted, and a defense mode was established. Finally, the expression of DEGs was validated by quantitative RT-PCR. Comparisons between compatible and incompatible responses will provide insight into the resistance mechanism and identify candidate defense or resistance genes for further study.

## Materials and Methods

### Plant Materials and Nematode Culture

The soybean breeding line 09-138 was developed by the Heilongjiang Academy of Agricultural Sciences (Hua et al., 2018). SCN race 4 (SCN4, HG type 1.2.3.5.6.7) and SCN race 5 (SCN5, HG type 2.5.7) were originally collected from the field and cultured from single cysts for more than 5 generations on the susceptible soybean variety Dongsheng1 in a greenhouse with 16 h of light and 8 h of darkness at 23–28°C, and then identified by race test and HG type indicator lines (Hua et al., 2018). Every other year, nematode HG types/races were reconfirmed without virulence change with time.

A nematode inoculum was prepared according to the method described by Huang et al. (2022). Plant root tissue and soil were collected 35-40 days after inoculation and put into a 2 L-beaker. The mixture was stirred vigorously with a glass rod for approximately 1 min and then precipitated for 10 s. The fluid of the supernatant was gently poured into 75/25 μm nested sieves. The mixture of cysts and root debris on the top of the sieves was rubbed with a rubber stopper to release eggs. Eggs on 25-μm sieve were rinsed with a high-pressure water faucet for 1 min and then with sterile water before collection. The collected eggs were then transferred onto six to eight layers of tissue paper supported by a metal screen on a hatching dish containing 3 mM ZnSO4 in sterile water for hatch at 28°C. J2s were then collected for inoculation after 3-4 days.

### Nematode Inoculation, Root Staining, and Root Preparation for RNA Extraction

Seeds of 09-138 were sterilized by soaking in 0.5% sodium hypochlorite for 20 min and rinsed with sterile water three times. Two seeds were sown in a black plastic pot (8 cm diameter × 12 cm depth) filled with autoclaved soil and sand at a ratio of 1:1. After 4 days, two seedlings were thinned to one in each pot. An eight-day seedling was inoculated with a 1-ml suspension containing 2,000 J2 of SCN4 or SCN5. Seedlings were inoculated with 1 ml water as control. The plants were maintained in a growth chamber at a 16-/8-h day/night regime, 28°C day/22°C night, and 50% relative humidity.

Collected roots were stained 3, 6, 8, 10, and 12 days after inoculation with acid fuchsin (Byrd et al., 1983). Nematode development inside the roots was observed, and roots and nematodes were photographed under an Olympus SZX16 dissecting microscope using the Cellsens Standard image software (Olympus Corporation, Japan).

To collect roots for RNA extraction, plant roots 8 days after inoculation were washed and rinsed thoroughly with water. Three roots from each treatment were wrapped together with aluminum foil as one replication (one sample). Three replications were made for each treatment. A total of 9 samples with SCN4- and SCN5-infection and control were collected. Immediately, each prepared sample was put in liquid nitrogen to freeze it and was kept at -80°C for RNA extraction and sequencing.

### RNA Extraction, cDNA Library Construction, and Nanopore Sequencing

Total RNA was extracted using RNAeasy Plant Mini Kit (Qiagen, United States), and RNase-free DNase (Qiagen) was used to remove DNA contamination in the total RNA. The concentration, purity, and integrity of the extracted RNA were measured with 1% agar gel (Thermo Fisher Scientific, United States) and Agilent 2100 Bioanalyzer (Agilent Technologies, United States). cDNA library construction started with 1 μg total RNA using a cDNA-PCR sequencing kit (SQK-PCS109) provided by Oxford Nanopore Technologies (ONT, Inc., United Kingdom) following the manufacturer’s instructions. Final cDNA libraries were added to FLO-MIN109 flow-cells and run on the PromethION platform at Biomarker Technology Company (Beijing, China) for sequencing. Experimental processes including sample quality testing, library building, library-quality testing, and library sequencing were performed in accordance with standard procedures provided by ONT.

### Raw Data Processing to Obtain Full-Length Transcriptome

Raw reads were subject to filtering with an average read quality score ≤ 7, and read length ≤ 500 bp, and ribosomal RNA mapped to the rRNA database was also discarded. Primer sequences on both ends of clean reads were searched to determine full-length, non-chimeric (FLNC) sequences. Detected FLNC transcripts were then mapped to soybean reference genome Williams 82.a2.v1^1^ with minimap2 (Li, 2018) to obtain FLNC clusters, and pinfish^2^ was applied to polish each cluster to attain consensus isoforms. All mapped reads were further collapsed using the cDNA_Cupcake package with a mini-identity of 90% and mini-coverage of 85%. When the redundant transcripts were collapsed, 5′ difference was not considered. The achieved transcripts were compared with known transcripts of reference genome Williams 82.a2.v1 utilizing gffcompare, and novel transcripts were identified in order to make supplementary genome annotation. Gene boundaries were modified, and transcripts with expressed levels ≤ 1 were filtered.

### Structure Analysis: Identification of Alternative Polyadenylation, Fusion Transcript, Alternative Splicing Events, and Microsatellite Markers

Alternative polyadenylation was identified through further analysis of FLNC by Transcriptome Analysis Pipeline from Isoform Sequencing (TAPIS) (Foissac and Sammeth, 2007). Multiple Expectation Maximization for Motif Elicitation (MEME) (Bailey et al., 2006) was used to analyze the 50-bp sequence upstream of the poly A site to detect FL motifs. The consensus sequence before remove-redundant analysis was used for fusion transcript analysis. Fusion transcript was defined under the following conditions: aligned to 2 or more sites; each site covers at least 5% of a transcript with a 1-bp minimum alignment length; the total length covers more than 95% of the total length of transcripts with at least 10k bp distance between the two sites.

Alternative splicing indicates the process of pre-mRNA treatment. Gene transcription generates pre-mRNAs with many splicing methods. Five types of alternative splicing events (3’ splice site, 5’ splice site, exon skipping, intron retention, and mutually exclusive exon) of transcripts were examined by employing the Astalavista software based on the alignment results of individual samples to the reference genome (Foissac and Sammeth, 2007). The MIcroSAtellite (MISA, an identification tool) software was used for SSR analysis, and transcripts below 500 bp were discarded.

### Coding Sequence Prediction, Long Non-coding RNA Identification, and Transcription Factor Detection From Novel Transcripts

Coding sequences (CDSs) were predicted with TransDecoder (v3.0.0; Haas et al., 2013) based on the ORF. LncRNA does not code for protein. Therefore, LncRNA in novel transcripts was predicted whether it had a coding potential by protein domain analysis including all four methods, Coding Potential Calculator (CPC) (Kong et al., 2007), Coding-Non-Coding Index (CNCI) (Sun et al., 2013), Coding Potential Assessment Tool (CPAT) (Wang et al., 2013) and Protein family (Pfam) (Finn et al., 2014). LncRNA target genes were predicted using two methods: first, depending on the location relationship between the differentially expressed lncRNA and adjacent mRNA (within 100k bp distance) expressed differentially; second, according to complementary base pairing between lncRNA and mRNA using the lncTAR tool (Li et al., 2015). TFs were detected with iTAK (Zheng et al., 2016).

### Quantification of Transcript/Gene Expression Levels and Differential Expression Analysis

Mapped full-length reads with > 5 match quality were chosen for quantification. Transcript or gene expression levels were measured in counts per million (CPM) (Zhou et al., 2014) and calculated by the following:

CPM = (read number matched the transcript)/(total read number matched referenced transcriptome) × 10^6^

The differential expression among the treatments was analyzed with DESeq2 (Anders and Huber, 2010) depending on a negative binary distribution model, consequently gaining DEGs or DETs. False discovery rate (FDR) was adjusted and controlled with the method of Benjamini and Hochberg (1995), and DEGs or DETs with log_2_fold change (FC) ≥ 2 and FDR < 0.01 were chosen. A heat map for DEGs in each group was developed using the pheatmap package in R (Version 1.0.12^3^).

### Functional Annotation and Enrichment Analysis of Differentially Expressed Genes/Transcripts

Functional annotation of the genes/transcripts was conducted by blasting with databases including NR (NCBI non-redundant protein sequences) (Deng et al., 2006), Swissprot (Apweiler et al., 2004), GO (Ashburner et al., 2000), Clusters of Orthologous Groups (COG) (Tatusov et al., 2000), euKaryotic Ortholog Groups (KOG) (Koonin et al., 2004), Pfam (Kanehisa et al., 2004), and KEGG (Mckenna et al., 2010).

Gene ontology enrichment analysis of DEGs or DETs was conducted using the GOseq R package-based Wallenius non-central hypergeometric distribution (Young et al., 2010). KEGG pathway enrichment analysis of DEGs or DETs was subject to the KEGG Orthology Based Annotation System (KOBAS) software (Mao et al., 2005). Protein–protein interactions (PPIs) for all detected DEGs were predicted using the STRING database^4^ and were visualized in Cytoscape (Shannon et al., 2003).

### Validation of Differentially Expressed Genes by Real-Time Quantitative Reverse Transcription-PCR

Samples leftover from full-length transcriptome sequencing was subject to qRT-PCR for verification of DEGs. Primers were designed by using the Primer Premier 5 software (Lalitha, 2000) and synthesized by Comate Bioscience Company Limited (Changchun, China). A total of 1 μg of treated RNA was used to synthesize the first-strand cDNA using FastKing gDNA Dispelling RT SuperMix (TIANGEN, China). PCR reactions were conducted in LightCycler^®^ 480 System (Roche Life Science, United States) with ChamQ Universal SYBR qPCR Master Mix (Vazyme Biotech Co., Ltd., Nanjing, China) following the manufacturer’s protocol. PCR was carried out in a 20-μl volume containing 100 ng cDNA (2 μl). PCR was performed as follows: initial denaturing for 10 min at 95°C, followed by 40 two-step cycles of 95°C for 10 s and then at 60°C for 1 min. The relative expression of tested genes was calculated with the –ΔΔCt method using ACTIN as a control. Three independent biological replicates and three technical repetitions were performed for all the experiments. The primers used are listed in Supplementary Table 1. The correlation between transcriptome sequencing and qRT-PCR was completed in Excel 2016, and independent-samples *t*-test was performed using SPSS 17.0.

## Results

### Differential Development of *Heterodera glycines* Race 4 and Race 5 Inside Roots of Lines 09-138 in Early Stages

**FIGURE 1:**
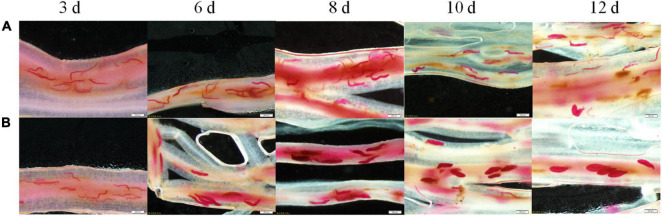
Development of soybean cyst nematode **(A)** race 4 (HG type 1.2.3.5.6.7) and **(B)** race 5 (HG type 2.5.7) inside the roots of soybean genotype 09-138 at 3, 6, 8, 10, and 12 days as indicated at the top of the image after inoculation with 2,000 J2. The roots were stained with acid fuchsin. Scale bar = 200 μm.

Various development stages of the two races, SCN4 (Figure 1A) and SCN5 (Figure 1B), were observed in roots. Soybean roots were penetrated by both nematode races, and there was no obvious difference in nematode size on day 3 days (Figure 1). On day 6, most of SCN5 developed to the J3 stage but SCN4 remained in the J2 stage. Nematodes had developed from J3 to late J4 stages in roots infected with SCN5 at 8, 10, and 12 days (Figure 1B) when compared with J2, J3, or a few early J4 stages in roots infected with SCN4 (Figure 1A), confirming 09-138 is resistant to SCN4 (cyst number/plant, 13 ± SE 2.7; female index, FI = 10) and susceptible to SCN5 (cyst number/plant, 119 ± SE 8.16; FI = 40) (Huang et al., 2022). More brown spots (hypersensitive response) around some nematode feeding sites were observed in resistant roots with SCN4 than in those with SCN5 (Figure 1A).

### Nanopore Full-Length Transcriptome Sequence Statistics and Redundant Remove of Transcript

Since an obvious difference in nematode development on day 8 was observed in 09-138, root samples from day 8 were used for full-length sequencing. An average of 6.1 Gbp of clean data for nine cDNA libraries was obtained with a range from 5.73 to 6.82 Gbp (Supplementary Table 2). The average N50 length was 1,287 bp with a mean length of 1,142 bp, an average max length of 11,276 bp (9,575-13,426 bp), and a mean quality value of 11 (Q11) (Supplementary Table 2). After rRNA was filtered out, an average of 5,296,377 clean reads (4,681,541-5,888,283) and an average of 4,258,467 FLNC read numbers (3,767,343-4,641,061) were generated with an average of 80.4% FLNC ratio (Table 1).

**TABLE 1:** Number of clean reads and full-length reads, and full-length percentage.

Treatment^a^	Number of clean reads (except rRNA)^b^	Number of full-length reads^c^	Full-length percentage(%)^d^
CK-1	5,592,903	4,437,097	79.33
CK-2	5,683,524	4,641,061	81.66
CK-3	5,399,629	4,311,944	79.86
SCN4-1	5,459,017	4,409,582	80.78
SCN4-2	5,888,283	4,631,268	78.65
SCN4-3	5,184,003	4,146,135	79.98
SCN5-1	4,727,932	3,785,053	80.06
SCN5-2	5,050,558	4,196,724	83.09
SCN5-3	4,681,541	3,767,343	80.47
Mean	5,296,377	4,258,467	80.43

*^a^CK-1, CK-2, and CK-3 represent control with water for three biological replications on soybean breeding line 09-138; Similarly, SCN4-1, SCN4-2, and SCN4-3 represent root samples infected with soybean cyst nematode race 4 (HG type 1.2.3.5.6.7) for three replications; and SCN5-1, SCN5-2 and SCN5-3 represent SCN race 5 for three replications. ^b^Number of clean reads (except rRNA): number of clean read sequences after filtering rRNA. ^c^Number of full-length reads: number of full-length sequences. ^d^Full-length percentage (%): percentage of number of full-length reads compared with clean reads.*

Finally, 65,038 redundant-removed transcript sequences containing 92,079,818 bp with an N50 length of 1,665 bp, mean length of 1,415 bp, and max-length of 7,285 bp were obtained through a merging of the consistent sequences (Supplementary Figure 1). After alignment with the reference genome and filtering with known annotations of the reference genome, 1,117 novel genes and 41,096 novel transcripts were identified. The consistent sequence of each sample was used for AS analysis.

### Structure Analysis of Alternative Polyadenylation Events, Transcript Fusion, Alternative Splicing Events, and SSR Prediction Revealed Soybean Structure Variation in Response to *Heterodera glycines* Race 4 and Race 5 Infections

**FIGURE 2:**
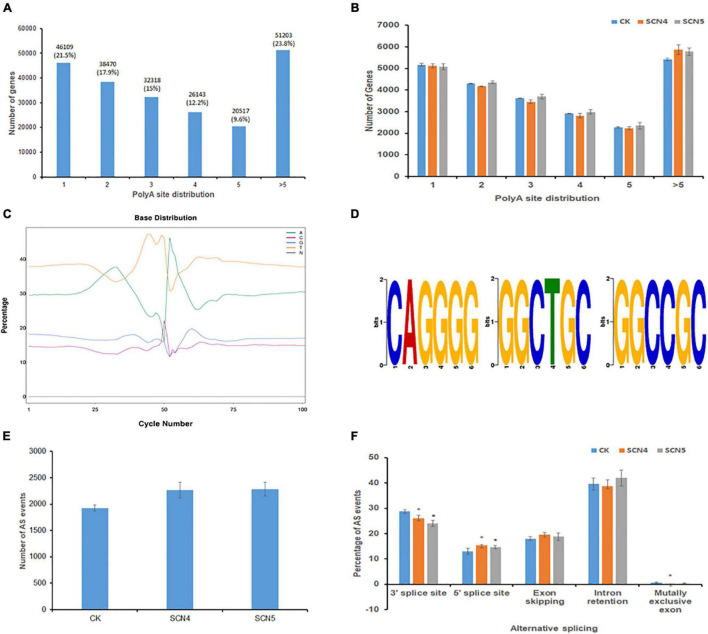
Alternative polyadenylation (APA) and alternative splicing (AS) event analysis on soybean genotype 09-138 infected with soybean cyst nematode race 4 (SCN4) and race 5 (SCN5), and water as the control (CK). **(A)** Distribution of total poly A sites of genes. **(B)** Comparison of poly A site distribution among CK, SCN4, and SCN5 treatments. **(C)** Base distribution (in%) at 50 bp upstream and downstream of the poly A site of all transcripts. **(D)** Identified APA motifs. **(E)** Total AS number for CK, SCN4, and SCN5. **(F)** Comparison of AS events among CK, SCN4, and SCN5 treatments.

Full-length sequencing can accurately identify the structure of transcripts. APA analysis based on FLNC displayed a total of 214,760 transcripts with various numbers of poly A sites. Among the three treatments, the greatest portion of 23.8% transcripts contained > 5 poly A sites, followed by 1 poly A site (21.5%), and the least with 5 poly A sites (9.6%) (Figure 2A). The number of transcripts in each poly A site distribution showed no significant difference (*P* > 0.05) among the three treatments (Figure 2B). In a more dissecting way, we found that SCN4 infection made soybean generate a significantly greater (*P* < 0.05) mean number (840 ± SE 26) of >10 poly A sites than SCN5 infection (734 ± SE 12) and the control (698 ± SE 26), indicating APA with more poly A sites might be involved in the incompatible reaction. Enrichment in T and A were detected in the upstream and downstream of the 50-bp position, respectively (Figure 2C). Three GC-rich APA motifs (CAGGGG, GGCTGC, and GGCCGC) were identified in the 50-bp sequence upstream of the poly A sites (Figure 2D). Fusion transcript screening before the remove-redundant analysis revealed 2-12 fusion transcripts for each sample. Similarly, SCN4 infection generated a total of 21 fusion transcripts, while only 14 fusion transcripts were found for SCN5 and the control (Supplementary Table 3), suggesting that fusion transcripts might be participating in defense response.

The Astalavista analysis of novel transcripts demonstrated that all the three treatments had no significant difference in the average number of AS events, 1,925 ± SE 54.6, 2,265 ± SE 145.8; and 2,287 ± SE 130.1 for the control, SCN4, and SCN5 treatments, respectively (Figure 2E), but that the AS events varied among the treatments. Based on the average percentage of AS events among the 9 libraries, there were 40.11% intron retentions, 26.28% alternative 3’ splice sites, 18.81% exon skipping, 14.34% alternative 5’ splice sites, and 0.46% mutually exclusive exons (Figure 2F). The numbers of AS events at alternative 3’ splice sites in both nematode-infected treatments were significantly lower (*P* < 0.05) than those in the control, while the numbers at 5’ splice sites were significantly greater (*P* < 0.05) than those in the control.

A total of 22,486 SSRs were identified from 61,900 novel transcripts containing a total of 90,765,603 bp (Supplementary Table 4). Three types of SSR with mono-, di-, and tri-nucleotide accounted for 98% of total SSRs. There were 1,718 SSRs present in compound formation (hybrid microsatellite, a distance of two SSRs < 100 bp) (Supplementary Figure 2 and Supplementary Table 4).

Thus, all the tested structure variations among the control, SCN4, and SCN5 treatments demonstrated that post-transcriptional modification might be involved in nematode resistance or susceptibility in soybean.

### Coding Sequences Prediction of Novel Transcripts

**FIGURE 3:**
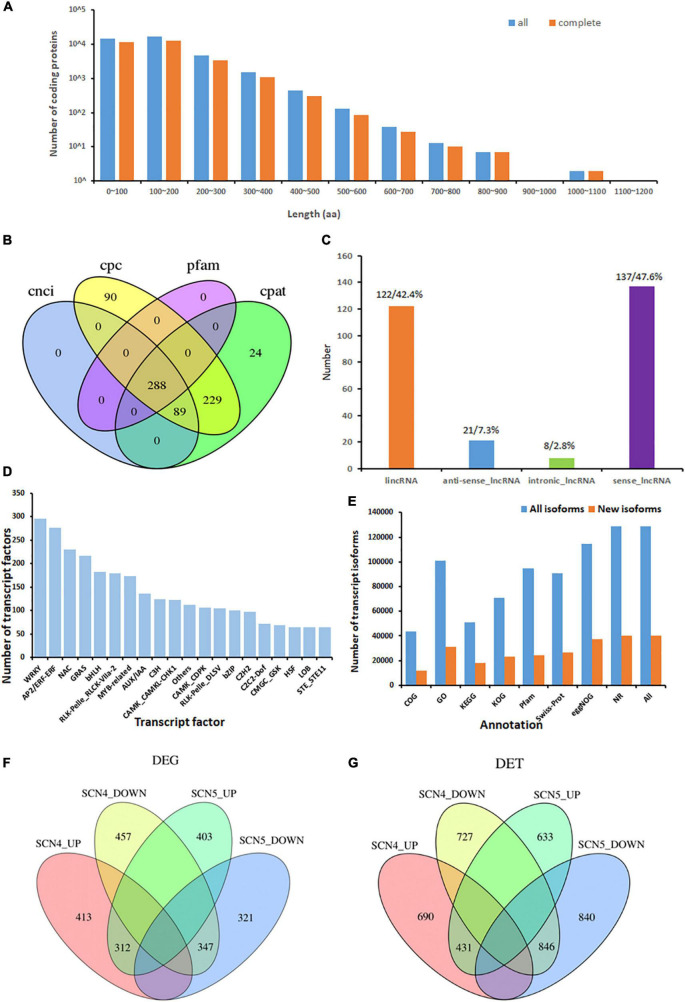
Isoform coding sequence length, lncRNA, and transcription factors from the novel transcripts identified from 9 soybean samples, and differentially expressed genes (DEGs) or transcripts (DETs) in soybean response to soybean cyst nematode race 4 (SCN4) and race 5 (SCN5) when compared with the control (CK). **(A)** Length distribution of all/complete coding protein sequences predicted (unit, aa, amino acid). **(B)** Number of lncRNAs identified with the CPC, CNCI, CPAT, and Pfam methods. **(C)** Percentage of the classification of lncRNA positions annotated with the reference genomes. **(D)** Distribution of top 20 enriched transcription factor types. **(E)** Number of all and novel transcript isoforms annotated by the COG, GO, KEGG, KOG, Pfam, Swiss-Prot, eggNOG, and NR databases. **(F,G)** Number of upregulated and downregulated DEGs and DETs between the CK-SCN4 and CK-SCN5 treatments.

A total number of 37,469 ORFs were obtained, including 28,759 complete ORFs. Approximately, 38 and 43% of all predicted CDS coding protein lengths were within 0-100 and 100-200 aa, respectively (Figure 3A). The corresponding complete CDS protein lengths are shown in Figure 3A.

### Long Non-coding RNA Associated With Defense Responsive Transcription Factors

Long Non-coding RNAs associated with plant growth, development, and stress responses have garnered widespread attention (Szcześniak et al., 2015; Urquiaga et al., 2021). In total, 288 lncRNAs were predicted by CPC, CNC, CPAT, and Pfam protein domain analyses; 24 and 90 of them were unique to the CPAT and CPC methods, respectively (Figure 3B); 47.6 (137) and 42.4% (122) of them were classified as sense_lncRNA and lincRNA (long intergenic non-coding RNA), respectively (Figure 3C). Antisense-lncRNA and intronic-lncRNA accounted for 7.3 (21) and 2.8% (8), respectively (Figure 3C). The number of cis-targeted genes (275) regulated by these lncRNAs was nearly four times greater than those (85) of trans-targeted genes. A lncRNA-Pfam protein domain analysis exhibited 164 transcripts matched to the Pfam database, including defense-responsive transcription factors WRKY, TIR, EF-hand, AUX/IAA, zf-RVT (zinc finger in the reverse transcript), zf-CCHC, BolA (bacterial stress-induced morphogen protein) and others, suggesting that lncRNAs may be involved in nematode stress response in soybean. Interestingly, 69 of 164 (42%) Pfam domains were extensin-like protein repeats from two novel transcripts, ONT.12398 (chr 9) and ONT. 12400 (chr 9), and 3 domains were extensin-like regions in ONT.14325 (chr 10). Extensins are a family of hydroxyproline-rich glycoproteins (HRGPs) in plant cell walls that can mediate resistance to viruses, bacteria, fungi, and nematodes (Deepak et al., 2010; Hirao et al., 2012). Especially, extensins identified in SCN syncytium formation (Ithal et al., 2007; Matsye et al., 2011) and nematode-related lncRNA co-expression with HRGP potentially mediating SCN infection (Khoei et al., 2021) consistently confirmed that altered cell wall composition with extensins may play roles in disease defense.

### Transcription Factor Prediction and Function Analysis of the Novel Transcripts

There were 5,337 TFs predicted, including 176 families of TFs (transcription factor, transcription regulator, and protein kinase) with a number ranging from 1 to 296 (Supplementary Table 5). The three treatments, the control, SCN4 and SCN5, contained 1,804, 1,787, and 1,746 TFs, respectively. The most abundant TFs were WRKY (296), AP2/ERF-ERF (APETALA2/Ethylene Responsive Factors, 276), NAC (NAM, ATAF, and CUC, 230), and GRAS (GAI, RGA, and SCA, 217) (Figure 3D).

Function annotation of transcripts obtained from the alternative splicing analysis exhibited 128,648 known and 40,090 novel transcript isoforms (Supplementary Table 6) and 56,865 known and 859 novel genes (Supplementary Table 7). The number of annotated transcripts in all and new isoforms is displayed in Figure 3E. Among them, NR annotation of species distribution indicated that 86.8, 8.9, 0.95, and 0.4% of the transcripts were aligned to *G. max*, *G. soja*, *Phaseolus vulgaris*, and *Medicago truncatula*, respectively. The GO-enrichment analysis showed that 13,483 out of 100,994 (13.5%) *G. max* isoforms and 4,350 out of 31,102 (14.0%) novel isoforms were associated with response to stimulus (Supplementary Figures 3A,B).

### Transcript Expression, Differentially Expressed Genes, and Differentially Expressed Transcripts

The CPM density distribution of transcript expressions of samples is displayed in Supplementary Figure 4A, and the dispersion degree of expression level distribution in a sample is shown with a boxplot in Supplementary Figure 4B. The evaluation of the Pearson correlation coefficient among the biological replicates indicated that the r range was from 0.682 to 0.963 (Supplementary Figure 4C). A clear separation of the control treatment from the SCN4- and SCN5- infected treatments is shown in principal component analysis (PCA) (Supplementary Figure 4D).

In total, 2,255 DEGs and 4,167 DETs were identified for all three comparisons; the upregulated and downregulated DEGs or DETs are listed in Table 2, and volcano plots for the DEGs and DETs are shown in Supplementary Figure 5. There were no overlapping DEGs (Figure 3F) or DETs (Figure 3G) found between CK-SCN4-up and CK-SCN5-down and vice versa. The annotated numbers of DEGs and DETs with functional annotation database are listed in Supplementary Table 8. Only 7 DEGs were found between the SCN4- and SCN5- treatments, including 3 DEGs identified in CK-SCN5, *Glyma.04G061500* (protein serine/threonine activity, GO:0004674), *Glyma.20G205700* (serine-type endopeptidase inhibitor activity, GO:0004867), and *Glyma.U029900* (function unknown, plasma membrane, GO:0005886), and 4 DEGs were identified in CK-SCN4, *Glyma.03G120700* (calmodulin binding, GO:0005516), *Glyma.10G093900*, *Glyma.11G099300* (a structural constituent of ribosome, GO:0003735), and *Glyma.19G125300* (calmodulin binding, GO:0005516).

**TABLE 2:** Number of differentially expressed genes (DEGs) and transcripts (DETs) between un-infected (CK) and *Heterodera glycines*-infected treatments (SCN4 and SCN5), and between SCN5 and SCN4.

	DEG			DET		
	Total number	Up-regulated	Down-regulated	Total number	Up-regulated	Down-regulated
CKvsSCN4	1,533	726	807	2,694	1,121	1,573
CKvsSCN5	1,383	715	668	2,750	1,064	1,686
SCN5vsSCN4	7	6	1	8	7	1

*SCN, soybean cyst nematode; vs.: versus.*

### Differentially Expressed Genes-Gene Ontology Annotation and Enrichment Analysis

**FIGURE 4:**
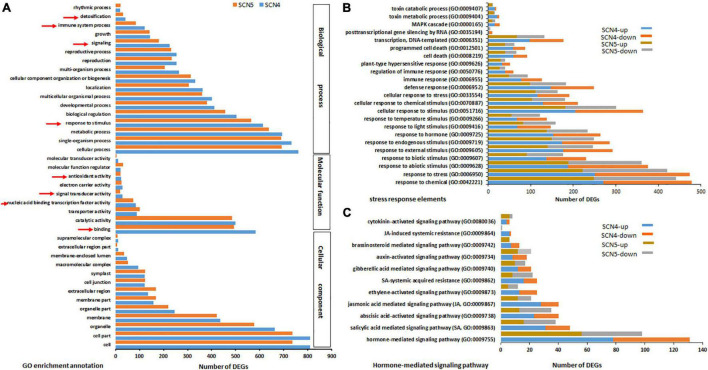
Top GO-enriched annotation of the differentially expressed genes (DEGs) **(A)** and comparison of DEGs with up-/downregulation associated with stress response elements **(B)** and hormones **(C)** in soybean 09-138 infected by soybean cyst nematode race 4 (SCN4) and race 5 (SCN5) when compared with the control (CK). The red arrowheads in A indicate stress response elements.

By GO annotation analysis, even though only 7 DEGs were found between the SCN4- and SCN5- infected soybean roots, the SCN4-treated roots displayed 986 DEGs, while the SCN5-treated roots showed 913 DEGs when each was compared with the control. The top GO classifications for both CK-SCN4 and CK-SCN5 treatments in BP, MF, and CC are displayed in Supplementary Figures 6A,B, respectively. The top three enriched BPs for both treatments were a response to growth hormone, auxin-activated signaling pathway, and multidimensional cell growth. The top three CCs were plant-type cell wall and intracellular membrane-bound organelle for CK-SCN4, cell part, intracellular part, and intracellular membrane-bound organelle for CK-SCN5. The top two enriched MFs for both CK-SCN4 and CK-SCN5 were quercetin 3-O-glucosyltransferase (GTF, EC:2.4) activity and coniferyl-alcohol GTF activity, and the third MF type was pectinesterase (PE, EC:3.1.1.11) activity for CK-SCN4 and quercetin 4’-O-GTF activity for CK-SCN5. Various numbers of enriched DEGs for BP, MF, and CC were observed between CK-SCN4 and CK-SCN5 (Figure 4A). Especially, the numbers of SREs (arrowhead pointing in Figure 4A) in CK-SCN4 are greater than those in CK-SCN5, e.g., response to stimulus (SCN4/SCN5, 613/565), signaling (225/181), immune system process (121/84), detoxification (41/32), and binding (582/493) (Figure 4A).

### *Heterodera glycines* Race 4 Infection-Induced More Stress Response Elements With More Upregulation Than *H. glycines* Race 5 Infection

In further analysis with BP annotation of SREs, a greater number of upregulated DEGs than that of downregulated ones was found in both CK-SCN4 and CK-SCN5 (Figure 4B and Supplementary Table 9). For example, the total number of the up-/down-regulated DEGs added together for each detected SRE was 8,780/6,662 for CK-SCN4 and 7,113/5,932 for CK-SCN5, indicating that more SRE genes were induced in the incompatible reaction than in the compatible reaction (Figure 4 and Supplementary Table 9). The BP response to stimulus included all kinds of biotic and abiotic stresses or stimuli (Supplementary Table 9), e.g., pathogens or pests, organic or inorganic chemicals, hormones, temperature, endogenous or external stimulus, defense response, cell death, hypersensitive response, immune response, gene silencing, and MAP kinases (Figure 4B).

Since the response to growth hormone was the first top enriched GO-BP for both treatments mentioned above, the response to each hormone was inspected and compared. Different responses to eight types of hormones were identified in both resistant and susceptible reactions compared with the control (Figure 4C and Supplementary Table 9). Although the hormones responded to infection with both nematode races, a greater number of upregulated DEGs than down-regulated ones were found in the resistant reaction to SCN4 in SA- (up/down, 31/17), JA- (28/12), abscisic acid- (ABA, 23/17), ET(13/12), gibberellic acid- (GA, 12/9), brassinosteroid- (BR, 7/6), and cytokinin- (CTK, 4/2) mediated signaling pathways and SA- (16/9) and JA- (6/1) induced systemic resistance except for auxin-activated signaling pathway (IAA, 8/10) (Figure 4C). On the contrary, in the susceptible CK-SCN5 reaction, a lower number of upregulated than downregulated DEGs were found in SA- (16/22), ABA- (13/22), and ET- (5/7) meditated signaling pathways, and SA- (8/14) or JA- (0/1) induced systemic resistance. These results suggested that more SREs with more upregulation were activated by the incompatible reaction with the SCN4 infection when compared with the SCN5 infection.

#### *Heterodera glycines* Race 5 Infection Induced More Differentially Expressed Genes With More Upregulated Genes Associated With Cell Wall Modification and Carbohydrate Biological Process Than *H. glycines* Race 4 Infection When Each Was Compared With the Control

**FIGURE 5:**
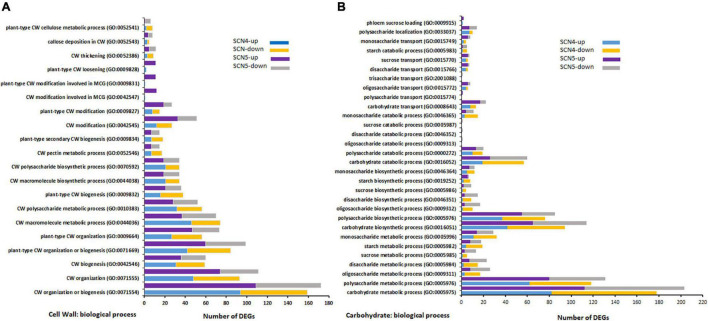
Top GO-enriched differentially expressed up-/down-regulated genes (DEGs) associated with cell wall (CW) modification **(A)** and carbohydrate biological process **(B)** on soybean breeding line 09-138 infected by SCN4 (soybean cyst nematode race 4) and SCN5 when compared with the control. The *x*-axis displays DEG number, and the *y*-axis represents the GO pathway.

Unsurprisingly, 23 (17 up/6 down) and 2 (2 up/0 down) DEGs for response to nematodes and syncytium formation were identified in the resistant soybean reaction to SCN4, respectively, while 34 (24 up/10 down) and 9 (9 up/0 down) DEGs for response to nematodes and syncytium formation were found in the susceptible reaction to SCN5, respectively. SCN syncytium formation requires plant cell wall modification, while cell wall modification involves multidimensional cell growth (SCN4/SCN5 DEGs: 38/48) which was the third BP-enriched element in both treatments. Therefore, all annotations involved in the cell wall were collected together, and a total of 28/26 GOs were annotated for CK-SCN4/CK-SCN5 (Figure 5A). A greater number of DEGs in CK-SCN5 than in CK-SCN4 was detected in cell wall organization or biogenesis (SCN5/SCN4: 172/159), cell wall modification (51/27), plant-type cell wall modification (27/15), cell wall modification involved in multidimensional cell growth (12/1), plant-type cell wall loosening (11/2), cell wall thickening (11/9), and callose deposition in the cell wall (8/5).

Syncytium formation requires carbohydrates as a nutrition sink for nematode feeding, and sugar transporters are active in syncytia through inter- and intracellular transport processes (Hofmann et al., 2007, 2009; Hofmann and Grundler, 2008). The greater DEG number associated with syncytium formation in CK-SCN5 than in CK-SCN4 denoted the difference in the carbohydrate metabolic process. Thus, the biological processes of carbohydrates and corresponding three major groups (sugars, oligosaccharides, and polysaccharides), including metabolic, biosynthetic, catabolic and transport processes, were examined and compared between CK-SCN4 and CK-SCN5 (Figure 5B). In total, 178, 94, 57, and 13 DEG in CK-SCN4 were involved in carbohydrate metabolic, biosynthetic, catabolic, and transport processes, respectively; 203, 114, 60, and 22 DEGs in CK-SCN5 were associated with the four processes, respectively. For all carbohydrate-associated biological processes, 739 DEGs with 317 up-/422 downregulation in CK-SCN4 and 870 DEGs with 467 up-/403 downregulation in CK-SCN5 were found (Figure 5B). Polysaccharides are major components of carbohydrate metabolic, biosynthetic, and catabolic processes. The lower number of upregulated DEGs (130) than downregulated DEGs (195) for CK-SCN4 was detected in all the groups of carbohydrate metabolic and biosynthetic processes; for CK-SCN5, the greater number of upregulated genes than downregulated genes were only found in polysaccharide metabolic (up/down: 80/51), and biosynthetic (up/down: 55/30) processes and monosaccharide biosynthetic (7/5) process (Figure 5B). Polysaccharides and monosaccharides were a major carbohydrate catabolic process, but polysaccharides displayed more upregulation than downregulation, and monosaccharides showed more downregulation in both CK-SCN4 and CK-SCN5 (Figure 5B). The transport process of carbohydrates demonstrated that oligosaccharides and monosaccharides were major transport formats; only 1 upregulated DEG for polysaccharide transport was detected in CK-SCN5 but not in CK-SCN4. These results suggested that the compatible reaction induced more upregulated expression in the carbohydrate biological process than the incompatible reaction.

### Kyoto Encyclopedia of Genes and Genomes Pathway Enrichment Analysis

The KEGG annotation showed that 273 and 292 unigenes were involved in 96 pathways in CK-SCN4 and 91 pathways in CK-SCN5, respectively (Supplementary Table 10). Two treatments shared 83 pathways; 13 and 8 unique pathways were detected in CK-SCN4 and CK-SCN5, respectively, e.g., SNARE interactions in vesicular transport (3 DEGs) and oxidative phosphorylation (4 DEGs) only in CK-SCN4 and N-Glycan biosynthesis (4 DEGs) and fatty acid degradation (1 DEG) only in CK-SCN5.

**FIGURE 6:**
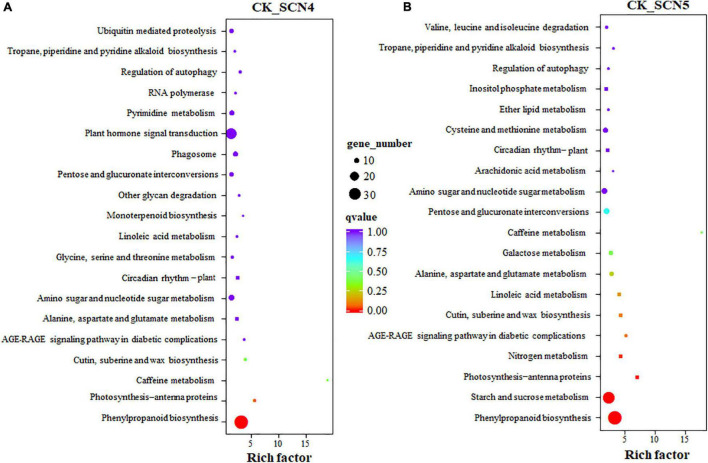
**(A,B)** KEGG pathway enrichment of DEGs (differentially expressed genes) in soybean treated with soybean cyst nematode race 4 (SCN4) and race 5 (SCN5) when compared with the control. Each circle represents one KEGG path, the ordinate axis represents the path name, and the horizontal axis is enrichment factor. Circle size suggests the number of enriched DEGs in the pathway, the larger the circle, the more DEGs.

Phenylpropanoid biosynthesis was the top enriched KEGG pathway for both CK-SCN4 (Figure 6A) and CK-SCN5 (Figure 6B). The following top 5 enriched KEGG pathways were photosynthesis-antenna proteins, caffeine metabolism, cutin, suberin and wax biosynthesis, and AGE-RAGE signaling pathway in diabetic complications for CK-SCN4 (Figure 6A), and starch and sucrose metabolism, photosynthesis-antenna proteins, nitrogen metabolism, and AGE-RAGE signaling pathway in diabetic complications for CK-SCN5 (Figure 6B). Among the top 20 enriched KEGG pathways, glycine, serine and threonine metabolism, monoterpenoid biosynthesis, other glycan degradation, phagosome, plant hormone signal transduction, pyrimidine metabolism, and ubiquitin-mediated proteolysis were unique for CK-SCN4; five metabolisms (nitrogen, galactose, cysteine and methionine, ether lipid, and inositol phosphate), and valine, leucine, and isoleucine degradation were unique for CK-SCN5 (Figure 6B). Interestingly, the KEGG pathway circadian rhythm-plant was enriched in the top 8th for CK-SCN4 and in the top 14th for CK-SCN5 (Figures 6A,B).

#### Phenylpropanoid Biosynthesis Pathway Was Enriched by Both Nematode Infections

**FIGURE 7:**
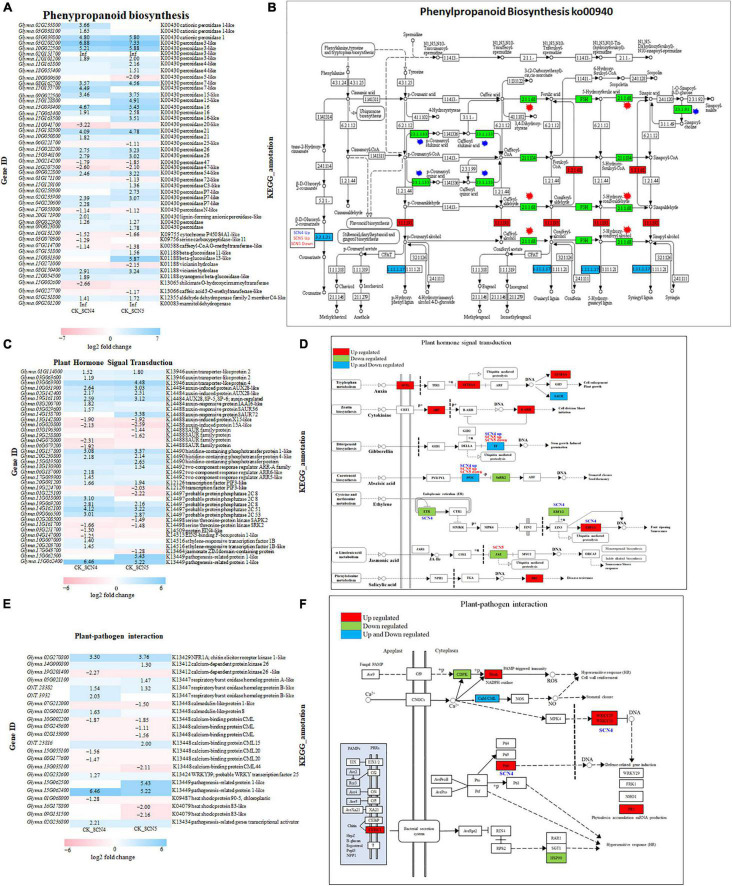
Gene expression and KEGG pathway in phenylpropanoid biosynthesis (https://www.genome.jp/pathway/map00940) **(A,B)**, plant hormone signal transduction (https://www.genome.jp/entry/map04075) **(C,D)** and plant-pathogen interaction (https://www.genome.jp/entry/map04626) **(E,F)** in soybean infected with SCN4 (soybean cyst nematode race 4) and SCN5. **(A,C,E)** represent the expression level of differentially expressed genes (DEGs) associated with the corresponding pathway for either CK (control) -SCN4 and/or CK-SCN5; white blank block (0) means no expression variations detected with Fold Change ≥ 2 and False Discovery Rate (FDR) < 0.05; the left side of the *y*-axis is gene ID, and the right side is the corresponding KEGG annotation. **(B,D,F)** are KEGG pathways; the blocks in red, green, and blue colors indicate DEGs in upregulation, downregulation, or both ways for both CK-SCN4 and CK-SCN5, respectively, except for the special note for DEGs only found in either SCN4/SCN5 or either up-/downregulation; in panel **(B)**, blue star represents the enzyme DEG found only in CK-SCN4, and the red star represents the enzyme DEG found only in CK-SCN5.

Both SCN4 and SCN5 infections induced phenylpropanoid biosynthesis, the top enriched KEGG pathway. Phenylpropanoid compounds are rich sources of metabolism in plants, providing precursors of lignin synthesis (Yao et al., 2021), while lignin is abundant in the cell wall, and it plays vital roles in plant defense in local or systematic required resistance (Dixon et al., 2002). Enzymatic reactions are considered key steps in the biosynthesis of major classes of phenylpropanoid compounds. Thirty-five KEGG orthologies of peroxidase (K00430, EC:1.11.1.7), the richest enzyme annotated in this pathway, were detected in either up-or down-regulation for both CK-SCN4 (up/down: 24/4) and CK-SCN5 (up/down: 23/6); 10 out of 35 *peroxidase* genes were unique for CK-SCN5, and 7 were unique for CK-SCN4 (Figures 7A,B). The peroxidase enzyme plays a role in the final step of the biosynthesis of lignin to produce p-hydroxyphenyl lignin, guaiacyl lignin, 5-hydroxyguaiacyl lignin, and syringyl lignin (Figure 7B). The gene expression levels of *peroxidase 3* (*Glyma.03G208200*, *Glyma.10G022500*) were increased by up to 5.2- to 7.3-fold after nematode infection when compared to the control. The SCN4 infection induced two downregulated DEGs: one gene, *Glyma.15G002600*, encoded enzyme shikimate O-hydroxycinnamoyltransferase (HST, EC:2.3.1.133), which catalyzes the reaction of two substrates, 4-coumaroyl-CoA (4-CCoA) and shikimate, to produce two products, CoA and 4-coumaroylshikimate (CSH); the other DEG, *Glyma.03G070300*, encoded serine carboxypeptidase-like 11 (SCPL11, EC:2.3.1.91), which can form sinapoylcholine (sinapine) and plays vital roles in abiotic and biotic stress responses, and growth and development as well (Xu et al., 2021). The SCN5 infection induced the downregulation of DEG *Glyma.04G227700* encoding caffeic acid 3-O-methyltransferase (COMT)-like (EC:2.1.1.68), a key enzyme regulating lignin synthesis (Trabucco et al., 2013) but not for the SCN4 infection. One gene (*Glyma.15G031300*) encoding the enzyme cyanogenic beta-glucosidase 13-like (EC:3.2.1.21) involved in cell wall lignification increased the expression level 5.87-fold by SCN5 infection (Figure 7A). Both nematode infections activated the upregulation of the DEG *Glyma.09G201200* encoding enzyme cinnamyl-alcohol dehydrogenase (CAD, EC:1.1.1.195) to catalyze the final step of monolignol biosynthesis, including p-coumaryl alcohol (H), coniferyl alcohol (G), and sinapyl alcohol (S), the main component of lignin (Figures 7A,B). These results indicated the expression variation of peroxidase enzyme, HST, SCPL, and other enzymes associated with lignin synthesis after nematode infection resulted in compatible and incompatible reactions by regulating plant cell wall modification.

#### Plant Hormone Signal Transduction, Plant–Pathogen Interaction, and MYB Transcription Factors Contributed to Defense Response

A similar number of DEGs were identified in both the plant hormone signal transduction pathway (SCN4/SCN5: 26/25) (Figures 7C,D) and the plant–pathogen interaction pathway (SCN4/SCN5: 12/14) (Figures 7E,F) but with various expressions in levels or gene types. As illustrated in Figures 7C,D, both nematode infections positively or negatively regulated the expression of the hormones (SA, JA, ET, GA, ABA, IAA, and CTK) but with qualitative and quantitative differences in soybean (Figure 7D). For example, the expression level of *PR1* (pathogenesis-related protein 1, *Glyma.15G062400*), which is involved in multiple pathways not only plant hormone signal transduction (SA) but also MAPK signaling and plant–pathogen interaction, was increased by more than 5-fold in soybean by the SCN4 (6.5-fold) and SCN5 (5.2-fold) infections when compared with the control. In the auxin pathway, which is linked to cell enlargement and plant growth, *AUX1* and *AUX/IAA* were upregulated, e.g., auxin transporter-like protein 4 (*Glyma.03G063900*) was positively regulated by both the SCN4 (4.1-fold) and SCN5 (4.5-fold) infections, and auxin-responsive gene *SAUR* (small auxin upregulated RNA) was both up- and downregulated (Figures 7C,D). Two transcription factors PIF3 (phytochrome-interacting factor 3)-like encoded by *Glyma.19G224700* and *Glyma.20G091200*, were identified in branch GA pathway as well as in circadian rhythm plant pathway, with the former being downregulated by both nematode infections and the latter being downregulated by the SCN5 infection. Three genes (*Glyma.09G066500*, *Glyma.19G069200*, and *Glyma.11G018000*) encoding PP2C (protein phosphatase 2C) in the ABA pathway were upregulated; the SCN4 infection induced an approximately 3-fold increase in the expression level of the three genes, and the SCN5 infection increased by 2.2- to 2.8-fold the expression level of the first two genes. JAZ (*jasmonate* zim domain) encoded by *Glyma.17G043700* in JA associated with stress response was detected only in CK-SCN5 with downregulation but not in CK-SCN4. On the contrary, DEGs encoding ETR (ethylene response sensor 2), EBF1 (EIN3-binding F-box protein 1), and ERF1 (ethylene-responsive transcription factor 1) in the ET pathway linked to fruit ripening and senescence were found only in resistant response to SCN4 but not to SCN5 when compared with the control (Figures 7C,D), indicating that the ET pathway played a role in defense response, and that JA expression was inhibited in susceptible response.

In the plant-pathogen interaction pathway, CERK1 (chitin elicitor receptor kinase 1, *Glyma.02G270800*) classified as PRRs in PTI, CDPK (calcium-dependent protein kinase), Rboh (respiratory burst oxidase) triggering reactive oxygen species (ROS) burst correlated to disease HR, CaM/CML (calmodulin/calmodulin-like protein) associated with HR, cell wall reinforcement and stomatal closure, and PR1 (*Glyma.15G062400*) linked to phytoalexin accumulation and miRNA production, were identified in both CK-SCN4 and CK-SCN5 but with various expression levels; downstream WRKY transcription factor *AtWRKY33* (*GmWRKY15*, *Glyma.02G232600*) and *Pti6* (PR genes transcriptional activator 6, *Glyma.02G236800*) connected to defense-related gene induction were significantly detected only in CK-SCN4 (Figures 7E,F). One gene, *Glyma.01G068000*, encoding HSP90 was detected with downregulation in CK-SCN4, but two other genes, *Glyma.09G131500* (*hsp83*) and *Glyma.16G178800* (*hsp83*), were negatively regulated with significance in CK-SCN5, suggesting that PTI was triggered initially by both nematode infections and that later, ETI was activated by the SCN4 infection.

In the KEGG analysis, 20 MYB or MYB-like transcription factors were detected; of these, 12 were found only in CK-SCN4 and 3 only in CK-SCN5 treatments, denoting that MYB transcription factors might regulate defense response. Three DEGs (*Glyma.03G261800*, *Glyma.16G017400*, and *Glyma.19G260900*) were associated with MYB-related transcription factor LHY involved in plant circadian rhythm as well, were negatively regulated after both SCN4 and SCN5 infections.

#### Starch and Sucrose Metabolism Pathway Regulated Susceptible Response to *Heterodera glycines* Race 5 and Chitinase I Was Upregulated in Defense Response to *H. glycines* Race 4

The top second KEGG enriched starch and sucrose metabolism pathway in CK-SCN5 treatment had 40 DEGs including 5 sucrose synthases, 11 pectinesterase-like or pectinesterase/pectinesterase inhibitors, 5 beta-glucosidase, and 4 alphas, alpha-trehalose-phosphate synthase [UDP-forming] and others, which are involved in plant growth and cell wall modification or cell wall lignification; these genes are necessary for syncytium formation. Of these, 8/9 and 16/19 up-/downregulated genes were identified for CK-SCN4 and CK-SCN5, respectively (Supplementary Figure 7A), e.g., *Glyma.15G223500* (pectinesterase/pectinesterase inhibitor 47) with a 5-fold increase in expression level after SCN5 infection, while upregulated *Glyma.03G216000* (pectinesterase/pectinesterase inhibitors 20) had 3.3-fold increased expression level in SCN4-infected roots, indicating that these DEGs played roles in the compatible or incompatible reaction. In the amino sugar and nucleotide pathway, 17/3 up-/down regulated DEGs were found, two of which had more than 4-fold significantly increased expression level, *Glyma.02G042500* encoding chitinase class I precursor and *Glyma.18G120700* encoding hevamine-A-like protein in SCN4-infected roots (Supplementary Figure 7B), suggesting that the two genes might contribute to defense response with SCN4 infection.

### Protein–Protein Interaction Analysis Revealed Valine-Glutamine-WRKY Interactions and Cytokinin Two-Component Response Regulator ARR-Glutaredoxins-BolA Interactions Might Be Involved in Defense Response

The PPI analysis among all the 2,255 DEGs indicated a ratio of 7,063/2,632 (2.7 ×) of protein-protein networks for CK-SCN4/CK-SCN5, including 866/194 activation (4.5 ×), 2715/1381 (2 ×) binding, 781/280 (2.8 ×) catalysis, 401/104 (3.9 ×) expression, 462/96 (4.8 ×) inhibition, 874/166 (5.3 ×) ptmod (post translational modification), and 964/411 (2.3 ×) reaction, indicating soybean resistant response to the SCN4 infection activated more protein–protein interactions than a susceptible response to the SCN5 infection, especially in post translational modification, inhibition, and activation.

**FIGURE 8:**
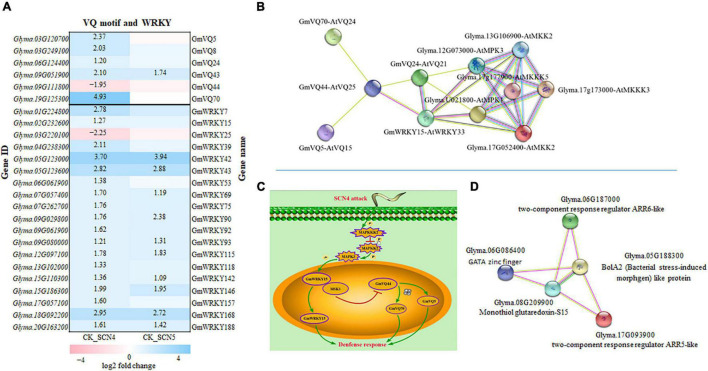
Gene expression of VQ (valine-glutamine motif) and transcription factor WRKY in soybean infected with soybean cyst nematode race 4 (SCN4) and SCN5 when compared with the control (CK), and protein-protein interactions. **(A)** All differentially expressed VQ and WRKY genes are displayed; white blank block (0) means no expression variations detected with Fold Change ≥ 2 and False Discovery Rate (FDR) < 0.05; the left side of the *y*-axis is gene ID and the right side is the corresponding gene name (Yu et al., 2016; Wang et al., 2019). **(B)** VQ-WRKY-MPK-MKK-MKKK interactions (MPK, mitogen-activated protein kinases). **(C)** Schematic model for VQ-WRKY interaction roles in defense response; SCN4 infection could regulate enzyme activities of MKKK, MKK, and MPK. One model is that MPK activation is able to release WRKY from the substrate MSK1 (GmVQ24) and function for plant defense; the other model is that the released MSK1 binding with GmVQ44 suppress *GmVQ44* expression, which may activate *GmVQ5* and *GmVQ70*, and consequently *GmVQ5/70* plays a role in plant defense. **(D)** ARR-BolA2-GRXS15 interactions; ARR-A, type A *Arabidopsis* response regulator; BolA2, (bacterial stress-induced morphogen)-like protein;GRXS15, monothiol glutaredoxin-S15. The lines in varied colors represent the type of interaction evidence (https://string-db.org).

Two genes, *Glyma.03G120700* (*GmVQ5*) and *Glyma.19G125300* (*GmVQ70*), were identified as significant DEGs with a 2.7-3.2-fold increase in CK-SCN4 than in CK-SCN5, encode calmodulin binding protein (CaMBP) (GO:0005516) carrying conserved VQ (Valine-Glutamine) motif (FxxhVQxhTG), which is capable of interacting with the transcription factor WRKY DNA-binding domain to play vital roles in stress response (Wang et al., 2014). Thus, the interactions between VQ and WRKY proteins encoded by DEGs were explored based on the Pfam domain; 6 (GmVQ5, GmVQ8, GmVQ24, GmVQ43, GmVQ44, and GmVQ70) out of 26 VQs and 19 out of 74 WRKYs annotated were detected in either CK-SCN4, CK-SCN5, or SCN5-SCN4 (Figure 8A). Interestingly, GmWRKY15, encoded by the upregulated *Glyma.02G232600*, homologous with *Arabidopsis* AtWRKY33 (WRKYGQK/WRKYGQK) in the plant–pathogen interaction pathway, was identified in CK-SCN4, which could interact with GmVQ24 (AtVQ21, *Glyma.06G124400*), and both were linked to GmVQ44 (AtVQ25, calcium-binding protein, *Glyma.09G111800*) with downregulation, which could bind with the two homologous CaM-binding proteins GmVQ5 and GmVQ70 (Figure 8B). VQ and WRKY numbers were designed according to Yu et al. (2016), Wang et al. (2019), respectively.

AtWRKY33 can interact with a VQ protein called mitogen-activated protein (MAP) kinase substrate1 (MSK1, AtVQ21), a substrate of MPK4 (MAP kinase 4), which can be activated in the presence of pathogen attack or flagellin, and then AtWRKY33 is released to induce the expression of phytoalexin deficient 3 (PAD3) in the nucleus, and thereby to increase defense response (Andreasson et al., 2005; Cheng et al., 2012). In addition, MPK3 and MPK6 can also interact with AtWRKY33 together, leading to an increase in phytoalexin-related gene expression (Alves et al., 2014). The overexpression of *AtWRKY33* resulting in decreased susceptibility to beet cyst nematode (*Heterodera schachtii*) (Ali et al., 2013) supports that *AtWRKY33* may be involved in soybean cyst nematode resistance. To find the kinase of the substrate GmVQ24 (MSK1) in the annotation data, all expressed MAP kinases were searched and no homologous AtMPK4 was found; interestingly, only two homologous MPK3s encoded by *Glyma.U021800* and *Glyma.12G073000*, each with a 1-fold increase in expression level in CK-SCN4, were identified to interact with GmWRKY15 and GmVQ24. Further searching of other DEGs encoding MAPKs exhibited that two genes encoding MAP kinase kinase 2 (MPKK2) had a 1.5-to 1.8-fold reduction in the expression level only in CK-SCN4 (Figure 8B). Two more MPKKKs encoded by *Glma.17G173000* and *Glyma.17G177900*, which could bind to MPK3 and MPKK2, were found with a 1.2-fold increase of gene expression in CK-SCN4 (data not shown). All these results demonstrated that MPKKK/MPKK/MPK/WRKY-VQ-CaMBPVQ might work together in soybean to defend against SCN4 infection. Based on these data, a model with a VQ–WRKY interaction in response to SCN4 infection was established with two possibilities, one is GmWRKY33 release by MPK3 activation or any other kinase to induce phytoalexin production, which activates plant defense as described in *Arabidopsis* (Alves et al., 2014); the other is GmVQ44 binding to GmVQ24 (MSK1) to suppress *GmVQ44* expression, which results in high expression of CaMBP *GmVQ5/70* to induce SCN resistance (Figure 8C).

Another interesting protein–protein interaction was detected in the plant hormone signal branch cytokinin pathway with two upregulated CK-SCN4 DEG genes (Figure 8D), *Glyma.06G187000* (*ARR6*, Log_2_FC = 2.2) and *Glyma.17G093900* (*ARR5*, Log_2_FC = 1.5), which are classified as two-component response regulator ARR-A (type A *Arabidopsis* response regulator) family composed of an inner membrane-spanning histidine kinase and a cytoplasmic response regulator to allow organisms to sense and respond to changes in environmental stimuli (Stock et al., 2000). In CK-SCN4, the ARR5-like and ARR6-like proteins were capable of interacting with a mitochondrial chaperone, monothiol glutaredoxin-S15 (GRXS15), and BolA2. GRXS15 encoded by the downregulated DEG *Glyma.08G209900* (Log_2_FC = -1.8), and BolA2 encoded by a downregulated DEG *Glyma.05G188300* (Log_2_FC = -1.6) could connect with the transcription factor GATA zinc finger (*Glyma.06G086400*, Log_2_FC = 1.7) (Figure 8C). Mitochondria GRAX15 was reported to play key roles in iron-sulfur protein maturation in the plant (Moseler et al., 2015) and a transcription regulator BolA2 containing a helix-turn-helix (HTH) motif for nucleic acid binding (Kasai et al., 2004). AtBolA3–GRXS17 interaction plays a key role in suppressing abiotic tolerance (Cheng et al., 2011; Qin et al., 2015). In a similar manner, inhibition of the gene expression of both *BolA2* and *GRAX15* with SCN4 infection resulting in resistance denoted that BolA2–GRAX15 interaction may negatively regulate soybean resistance to SCN4. The function of BolA2–GRAX15 interaction has not been reported yet, but it is known that BolA2 is nucleon-cytoplasmic and interacts with GRAX15 (Couturier et al., 2014). Furthermore, BolA2 was linked to the lncRNA detected above. Therefore, the interactions of ARR5/6-GRAX15-BolA2-GATA might indicate a new complex interaction partaking in plant defense in response to SCN4 infection.

### Validation of Differentially Expressed Genes by QRT-PCR Assay

**FIGURE 9:**
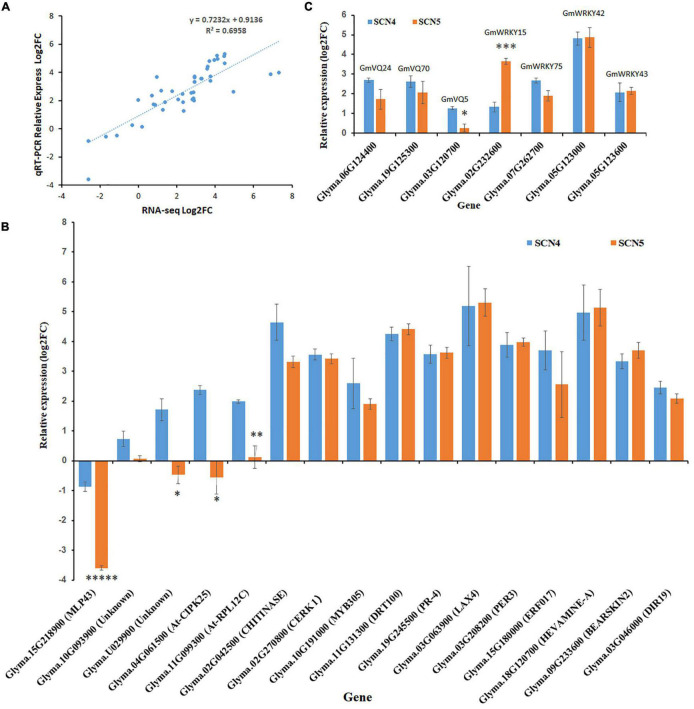
Validation of differentially expressed genes (DEGs) by qRT-PCR. **(A)** Correlation of DEG expression levels between RNA-seq and qRT-PCR. **(B)** Relative expression of *VQ* and *WRKY* genes. **(C)** Relative expression of 16 other DEGs. For panels **(B,C)**, the *x*-axis displays different genes and gene annotation, and the *y*-axis is relative expression level (log_2_FC-fold change). Relative expression level was calculated with the –ΔΔCt method using the *actin* gene as an endogenous control. The error bar stands for the SE. The asterisks represent significant difference by *t* test (*p < 0.05; ^**^p < 0.01; ^***^p < 0.005; and ^*****^p < 0.005).

The expression levels of 23 DEGs obtained from full-length transcriptome sequencing were compared with those obtained from qRT-PCR. The expression correlation (R^2^) between full-length-seq and qRT-PCR was up to 0.6958 (Figure 9A), and the comparison of relative expression levels between SCN4 and SCN5 demonstrated that the qRT-PCR data almost matched with the full-length-seq data (Figure 9B). The relative expression levels of four *WRKY* and three *VQ* genes are also displayed in Figure 9C.

## Discussion

### Full-Length Transcriptome Analysis: A Powerful Tool to Analyze Transcriptional and Post-transcriptional Regulation

In this study, full-length transcriptome analysis on soybean was first conducted to compare responses of the same soybean genotype incompatible and compatible to different *H. glycines* races. Obviously, the advantages of full-length transcriptome sequencing include high-cost performance, high throughput, no GC specificity, and base bias, long sequencing reads, accurate quantification at the transcriptome level, accurate identification of structure characteristics (e.g., AS, fusion gene, APA) without breaking sequences or gene structures, and DEG/DET analysis at once. Compared to NGS, ONT needs less read numbers to cover the same amount of transcripts. For example, an average of 3-5 × greater number of clean reads was obtained by NGS (Zhang et al., 2017; Neupane et al., 2019; Miraeiz et al., 2020) when compared with the average number of clear reads in this study. In addition, the identified novel genes can generate new AS events.

The change in numbers of AS events at 5’ splicing sites or at 3’ splicing sites after both nematode infections and various transcripts among the three treatments indicated that the nematode infections did trigger differential AS events to generate multiple transcripts that might result in plant defense or susceptibility. A few cases have been reported that AS regulates plant stress responses and/or adaptations (Bedre et al., 2019; John et al., 2021; Martín et al., 2021). For instance, the tobacco gene *N* resistant to *tobacco mosaic virus* (TMV) can be alternatively spliced to produce two transcripts that are required to contribute to complete resistance to TMV (Dinesh-Kumar and Baker, 2000). AS regulates ABA and light signaling pathways to coordinate plant growth and stress response (Thiruppathi, 2020).

Interestingly, we found that more abundant APA events per gene were identified with > 5 poly A sites (23.8%) than those in 1-5 poly A sites in soybean, while 2 poly A sites were found as the most abundant APA event distribution in sorghum (Chakrabarti et al., 2020), and 1 poly A site for the tree species *Liriodendron chinense* in the magnolia family (Tu et al., 2021). The poly A site distribution in plants can be shifted by abiotic stresses (Yan et al., 2021), and novel stress-specific cis-elements in intronic poly A sites may contribute to abiotic stresses (Chakrabarti et al., 2020). In this study, nematode infection increased the > 5-poly A site distribution than control, especially, the > 10 poly A sites in the incompatible reaction further confirmed that APA may be involved in nematode stress response. Generally, stress-responsive APA plays a key role in regulating abiotic stress and biotic stress through crucial stress-responsive genes and pathways (Ye et al., 2019; Chakrabarti et al., 2020; Yan et al., 2021). For instance, Ye et al. (2019) found that APA in rice responds to heat stress tolerance as a negative regulator, to Cd stress by regulating DNA repair and cell wall formation, and to disease stress (bacterial blight, rice stripe virus, and rice blast) by regulating chlorophyll metabolism. Comparisons of APA sites linked with annotated genes or pathways between control and nematode-infected treatments will uncover the APA function in soybean associated with nematode resistance or susceptibility. Furthermore, the difference in the fusion transcripts and lncRNAs among the treatments demonstrates that full-length transcriptome sequencing is a powerful tool to analyze post-transcriptional modification.

### Plant Hormone Signal Transduction and Plant–Pathogen Interaction Implicating Plant Defense Response

Both the GO and KEGG enrichment analyses consistently confirmed that stress response elements and associated pathways (plant hormone signal transduction and plant–pathogen interaction) contribute to plant innate immune response to SCN. Surprisingly, when all the DEGs in these groups/pathways identified in this study were compared with those listed by Zhang et al. (2017), Miraeiz et al. (2020), only 1-3 overlapping genes were found; the only DEG, *Glyma.11G207000*, encoding leucine-rich repeat-containing protein was found in all three studies. The difference might be caused by various SCN–soybean interaction systems and be affected by inoculation time, genotypes, nematode types, and possible nematode inoculation density. For example, a transcriptome analysis was conducted as early as 8 h post inoculation with HG type 0 on soybean Peking, *G. soja* PI 468916, Fayette, and Williams 82 by Miraeiz et al. (2020), and later sedentary phase at 3, 5 and 8 days (pooled samples) with HG type 2.5.7 on two *G. soja* genotypes by Zhang et al. (2017), and 8 days with HG Type 2.5.7 and HG Type 1.2.3.5.6.7 on the same genotype 09-138 in this study. Most transcriptome profiling studies are examined within 2-10 days since SCN feeding site establishment generally required 48 h after inoculation, and syncytium formation and syncytium collapse happens within 2-10 days after inoculation in resistance response (Klink et al., 2007; Kandoth et al., 2011).

Although uniquely differential expression genes to SCN4 or SCN5 infection were identified on 09-138, most DEGs were expressed in both resistant and susceptible reactions with only a small difference (Figure 7), indicating qualitative and quantitative traits. For example, in the plant–pathogen interaction pathway, upstream PPR-CERK1 and downstream Ca^2+^-dependent signal components CDPK, Rboh (triggering reactive oxygen burst, ROS), CaM/CML, HSP, and PR1 were all identified in both compatible and incompatible roots, and downstream WRKYs and Pti6 with upregulation were only detected in the incompatible response (Figures 7E,F), while these components are involved in PTI and/or ETI activity. In the plant-hormone pathway, all phytohormones except for brassinosteroid were activated with 1-4 key DEGs, while the phytohormone networks of the JA, ET, and SA signaling pathways are required for PTI and ETI as well (Cui et al., 2015). Naveed et al. (2020) found that both resistant and susceptible plants showed almost identical transcriptome responses, but that the resistant plants achieved high-amplitude transcriptional reprogramming several hours earlier than the susceptible plants through a defense phytohormone signaling network. Here, we only detected one time point in the later infection stage, and a time series examination will reveal more about how the pathways work together to defend against nematode attacks. The PTI-ETI continuum concept with crosstalk between PTI-ETI signal components effectively activating plant immune responses has the increasing attention of biologists (Mine et al., 2018; Naveed et al., 2020; Yuan et al., 2021), and this concept may also be applicable for SCN–soybean interaction based on the identified signal components in these pathways. In addition, surprisingly, even though 09-138 contained the Peking-*rhg1a* locus, there were not any DEGs found in that region, suggesting an alternative resistance mechanism in 09-138.

### Transcription Factor Functional Protein–Protein Interactions in Soybean Defense Response

Transcription factors as transcriptional regulators function by binding to the promoter region of target genes and regulating plant response to environmental stress, e.g., altering the expression of cascades of defense genes (Chen et al., 2002; Alves et al., 2014). The novel transcripts identified exhibited more than 5,000 TFs, with the top 5 being WRKY, AP2/ERF-ERF, NAC, GRAS, and bHLH. WRKY TFs, as the largest family of transcriptional regulators, were consistently identified in almost all transcriptome analyses of SCN infection (Ithal et al., 2007; Kandoth et al., 2011; Mazarei et al., 2011; Wan et al., 2015; Zhang et al., 2017; Song et al., 2019; Jiang et al., 2020; Miraeiz et al., 2020). The WRKY family, associated with stress responses in soybean, has been examined, e.g., in response to soybean rust disease (*Phakopsora pachyrhizi*) (Bencke-Malato et al., 2014), salt stress (Yu et al., 2016), dehydration and salt stress (Song et al., 2016), and soybean cyst nematode (Yang et al., 2017). However, 19 WRKY-DEGs to SCN4 or SCN5 identified in this study were not found in previous studies for SCN infection (Yang et al., 2017; Zhang et al., 2017; Miraeiz et al., 2020), indicating that these identified WRKY factors may be specific to the line 09-138 or the nematode races used in this study.

WRKY factors in plants are divided into five groups (I, IIa + IIb, IIc, IId + IIe, and III), and the interaction between the WRKY domain and its partner domain (e.g., VQ) is involved in signaling, transcription, and other important biological processes (Chi et al., 2013; Alves et al., 2014). The WRKY–VQ interaction has been well-studied in *Arabidopsis*. As mentioned above, AtWRKY33 (group I)/MSK1 interaction activates plant defense gene expression (Andreasson et al., 2005; Qiu et al., 2008; Alves et al., 2014). AtWRKY25, a homolog of AtWRKY33, is also able to interact with MSK1 and MPK4 in the absence of a pathogen (Cheng et al., 2012). Additionally, AtWRKY33 is able to interact with two other VQ proteins, AtSIB1 (sigma factor binding protein 1, AtVQ23) and AtSIB2 (AtVQ16), in the nucleus to activate resistance to a necrotrophic pathogen, *Botrytis cinerea* (Lai et al., 2011). In this study, the lack of homologous MPK4 indicates a possible alternate interaction mechanism participating in nematode defense response. In the soybean–pest interaction system, only one GmVQ58 was identified as a negative regulator for soybean resistance to the common cutworm (*Spodoptera litura* Fabricius), and GmVQ58 could interact with GmWRKY32 (Li X. et al., 2020).

The CaM family is composed of ubiquitous Ca^2+^-binding proteins or calcium sensor proteins, which can play a key role in cellular signaling cascades coupling various environmental stimuli (Zeng et al., 2015). For instance, AtCaMBP25/AtVQ15 was found as a negative effector regulating osmotic stress tolerance during seed germination and seedling growth (Perruc et al., 2004). CaM-mediating signaling can regulate ROS homeostasis directly and indirectly (Zeng et al., 2015). Here, we found DEGs *CaM/CML* in the plant-pathogen pathway and two *CaMBPGmVQ5/VQ70s*, which directly or indirectly interact with other VQs and WRKYs to form complex GmVQ5/70-GmVQ44-GmWRKY15-GmVQ24 (MSK1) (Figure 8B). Only the incompatible reaction could activate GmVQ5/70, GmWRKY15, and GmVQ24, and suppress the connector GmVQ44, indicating that VQ–WRKY interactions partake in plant defense response to SCN4. Additionally, the high expression level of *GmWRKY15* increasing SCN resistance is consistent with that the homologous *AtWRKY33* overexpression decreasing *H. schachtii* susceptibility (Ali et al., 2013).

### Top Enriched Phenylpropanoid Biosynthesis Pathway Involved in Both Resistant and Susceptible Response

The phenylpropanoid pathway has been considered a ubiquitous defense response against pathogens including nematodes, and this pathway is always enriched in SCN–soybean interaction (Edens et al., 1995; Dixon et al., 2002; Zhang et al., 2017; Li et al., 2018; Singh et al., 2019; Miraeiz et al., 2020). Approximately 75% of DEGs in this pathway were peroxidase, and more than 80% of the enriched peroxidase genes could be induced after nematode infection either in susceptible or in resistant response, which is matched with previous reports (Miraeiz et al., 2020), suggesting that peroxidase plays a central role in response to nematode attack. Peroxidase contributes to plant defense by modifying the cell wall composed of lignin, suberin, feruloylated polysaccharides, and HPRG (extensins), enhancing ROS production and phytoalexin productions (Pandey et al., 2017). The key enzymes linked to lignin biosynthesis and uniquely expressed in the incompatible (e.g., HST, *Glyma.15G002600*) or compatible (e.g., beta-glucosidase, *Glyma.15G031300*) reaction will be potentially targeted genes for further studies to elucidate plant defense or pathogenesis.

### Carbohydrate Biological Process and Cell Wall Modification Roles in Soybean Cyst Nematode Susceptibility

SCN5 infection-induced more DEGs with more upregulated genes in cell wall modification and carbohydrate metabolic process than SCN4 infection but with some quantitatively overlapping DEGs, indicating unique and accumulative DEGs in carbohydrate biological process including metabolism, biosynthesis, catalyst and transport work together to make SCN susceptibility or resistance. Miraeiz et al. (2020) also reported that susceptible W82 showed less responsive genes, more downregulated genes in carbohydrate metabolism and transport proteins, and some overlapping genes with the resistant genotype 8 h post inoculation. Nevertheless, again, there are less common DEGs found between 8 h post inoculation (Miraeiz et al., 2020) and 8 days (this study), a beta-glucosidase was downregulated at 8 h but upregulated at 8 days in this study, suggesting spatially and temporally differential expression. In the starch and sucrose metabolism pathway, sucrose synthase, hexokinase, beta-amylase, and polygalacturonase were suppressed more, and beta-glucosidase, beta-fructofuranosidase, and galacturonosyltransferase were induced more in the compatible interaction than in the incompatible interaction, proving the complexity for nematode pathogenicity. Hofmann et al. (2007) demonstrated that sucrose supply to *H. schachtii*-induced syncytia depends on the apoplasmic pathway in the early stage during syncytium formation and on the symplasmic pathway in the later stage when syncytia are linked to the phloem. The change of a series of enzymes during a metabolic process may explain various enzyme expression patterns in different SCN–soybean interaction systems both spatially and temporally.

## Conclusion

In conclusion, this study represents for the first time a full-length transcriptome sequencing comparison between compatible and incompatible reactions on the same soybean genome to *H. glycine*. Stress response elements, plant pathogen interaction pathway, plant hormone signaling transduction pathway, and transcription factors contributed to plant immune response. The related genes associated with cell wall modification and carbohydrate metabolism played critical roles in nematode susceptibility. The phenylpropanoid biosynthesis pathway was enriched by two nematode infections. For the first time, a model of WRKY–VQ interaction resulting in plant defense response to nematode infection in an incompatible reaction was established. The identified AS events, APA, and lncRNA will provide insights into the function of post transcriptional modification during plant–nematode interaction. The knowledge of SCN–soybean interaction will aid us to understand the evolution of resistance and susceptibility, and further functional studies will help to explore new control strategies against nematodes.

## Data Availability Statement

The datasets presented in this study can be found in online repositories. The name of the repository and accession number can be found below: NCBI; PRJNA803218.

## Author Contributions

CW and CL conceived and designed the study, and wrote, reviewed, and edited the manuscript. MH, YJ, RQ, DJ, and DC performed the laboratory work and conducted the data analysis. MH and YJ wrote the original draft. ZT was a curator of plant material. CW and MH validated the data and analysis. All authors read and approved the final manuscript.

## Conflict of Interest

The authors declare that the research was conducted in the absence of any commercial or financial relationships that could be construed as a potential conflict of interest.

## Publisher’s Note

All claims expressed in this article are solely those of the authors and do not necessarily represent those of their affiliated organizations, or those of the publisher, the editors and the reviewers. Any product that may be evaluated in this article, or claim that may be made by its manufacturer, is not guaranteed or endorsed by the publisher.

## Footnotes

1
https://phytozome.jgi.doe.gov/

2
https://github.com/nanoporetech/pinfish

3
https://CRAN.R-project.org/package=pheatmap

4
http://string-db.org

## Supplementary Material

Supplementary Table S1 Primer sequences for qRT-PCR.

Supplementary Table S2 Statistics of clean data.

Supplementary Table S3 All fusion gene list for different samples.

Supplementary Table S4 Summary of SSR analysis.

Supplementary Table S5 All identified transcription factors.

Supplementary Table S6 Novel isoform functional annotation.

Supplementary Table S7 Novel gene functional annotation.

Supplementary Table S8 Number of annotated DEGs and DETs.

Supplementary Table S9 Top DEG-GO annotation of stress response element comparison between CK vs SCN4 and CK vs SCN5.

Supplementary Table S10 Top DEG-KEGG pathway comparison between CK vs SCN4 and CK vs SCN5.
