# Supplementary material for: Full-Length Transcriptional Analysis of the Same Soybean Genotype With Compatible and Incompatible Reactions to Heterodera glycines Reveals Nematode Infection Activating Plant Defense Response
Source: Front Plant Sci. 2022 May 18;13:866322. doi: 10.3389/fpls.2022.866322 (PMC9158574; doi:10.3389/fpls.2022.866322)
Supplement: Supplementary Table S1 — Primer sequences for qRT-PCR. [file Data_Sheet_1.ZIP › Supplementary tables/All supplementary Table list.docx]

All supplementary Table list:

Table S1. Primer sequences for qRT-PCR.

Table S2. Statistics of clean data.

Table S3. All fusion gene list for different samples.

Table S4. Summary of SSR analysis.

Table S5. All identified transcription factors.

Table S6. Novel isoform functional annotation.

Table S7. Novel gene functional annotation.

Table S8. Number of annotated DEGs and DETs.

Table S9. Top DEG-GO annotation of stress response element comparison between CK vs SCN4 and CK vs SCN5.

Table S10. Top DEG-KEGG pathway comparison between CK vs SCN4 and CK vs SCN5.
