# Supplementary figures and images for: Full-Length Transcriptional Analysis of the Same Soybean Genotype With Compatible and Incompatible Reactions to Heterodera glycines Reveals Nematode Infection Activating Plant Defense Response
Source: Front Plant Sci. 2022 May 18;13:866322. doi: 10.3389/fpls.2022.866322 (PMC9158574; doi:10.3389/fpls.2022.866322)

## Slide 1
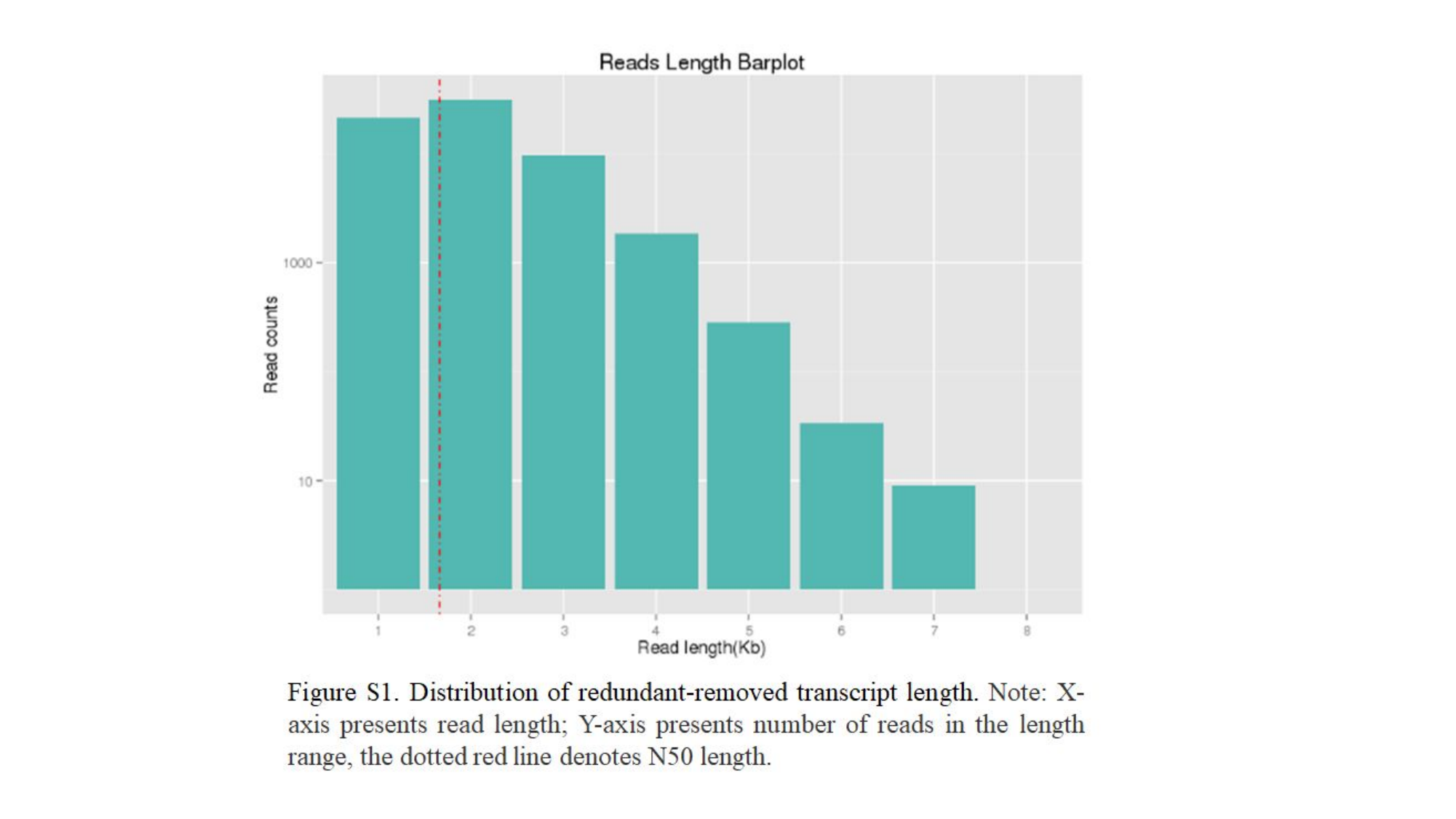

## Slide 2
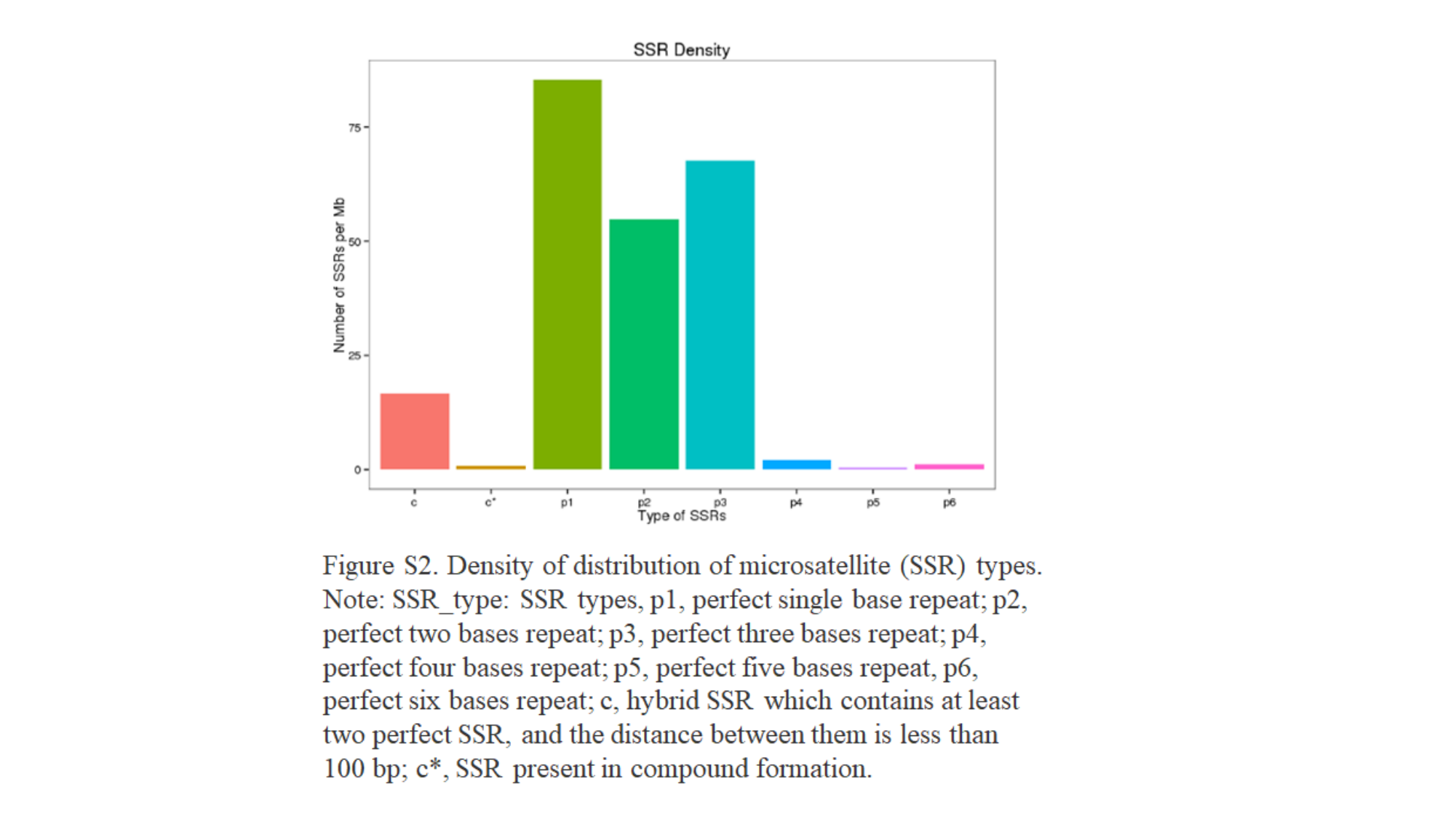

## Slide 3
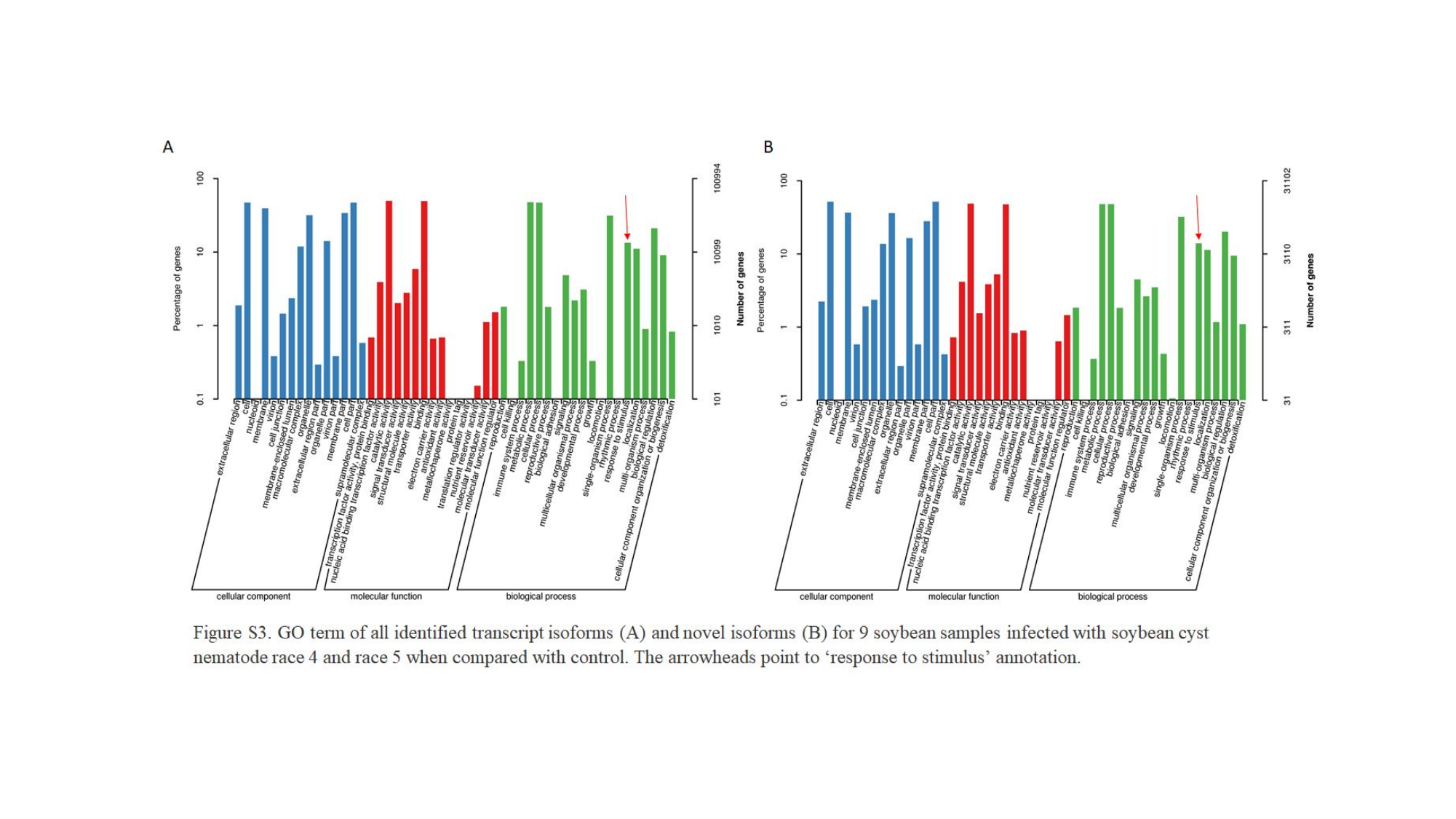

## Slide 4
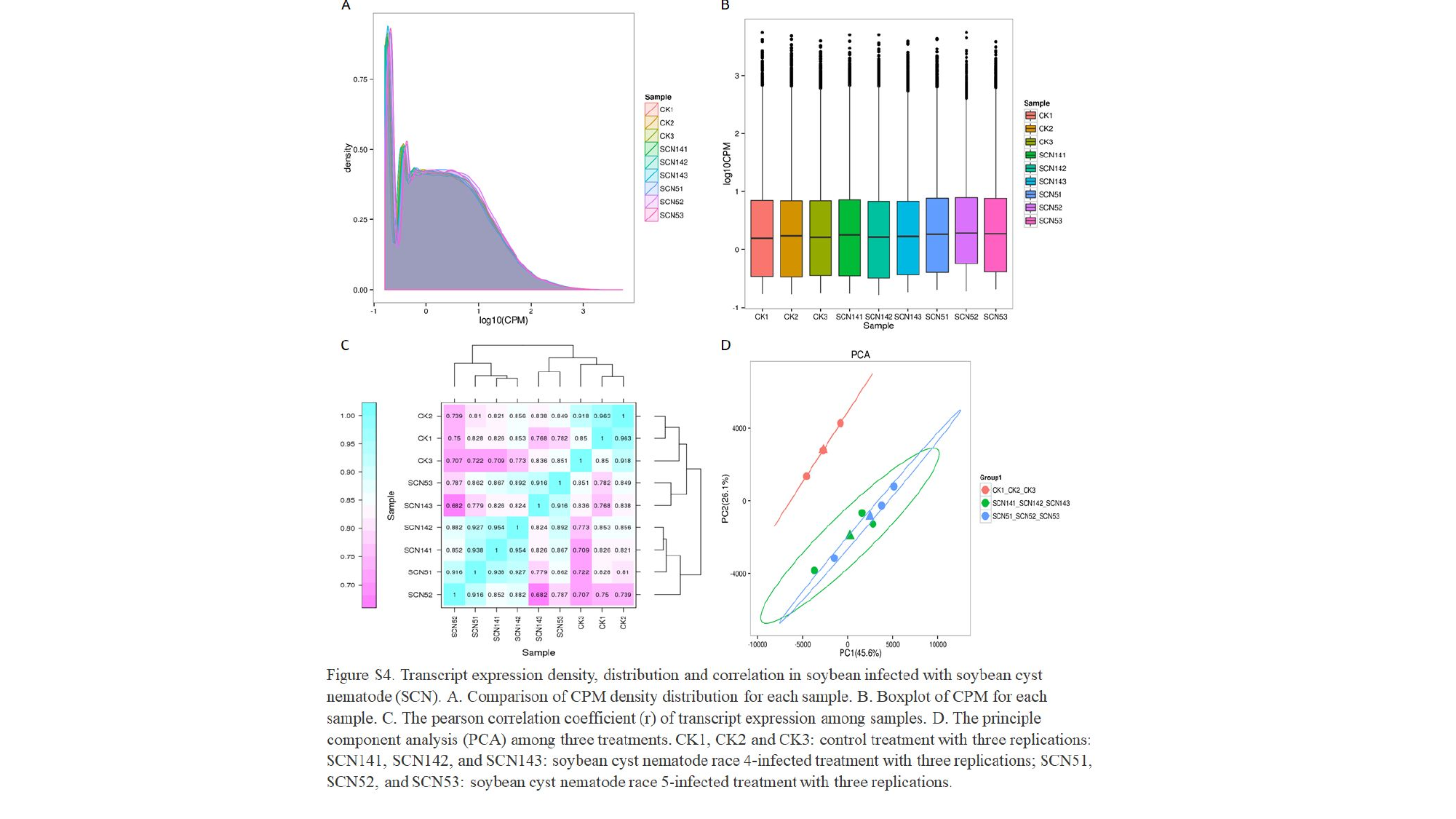

## Slide 5
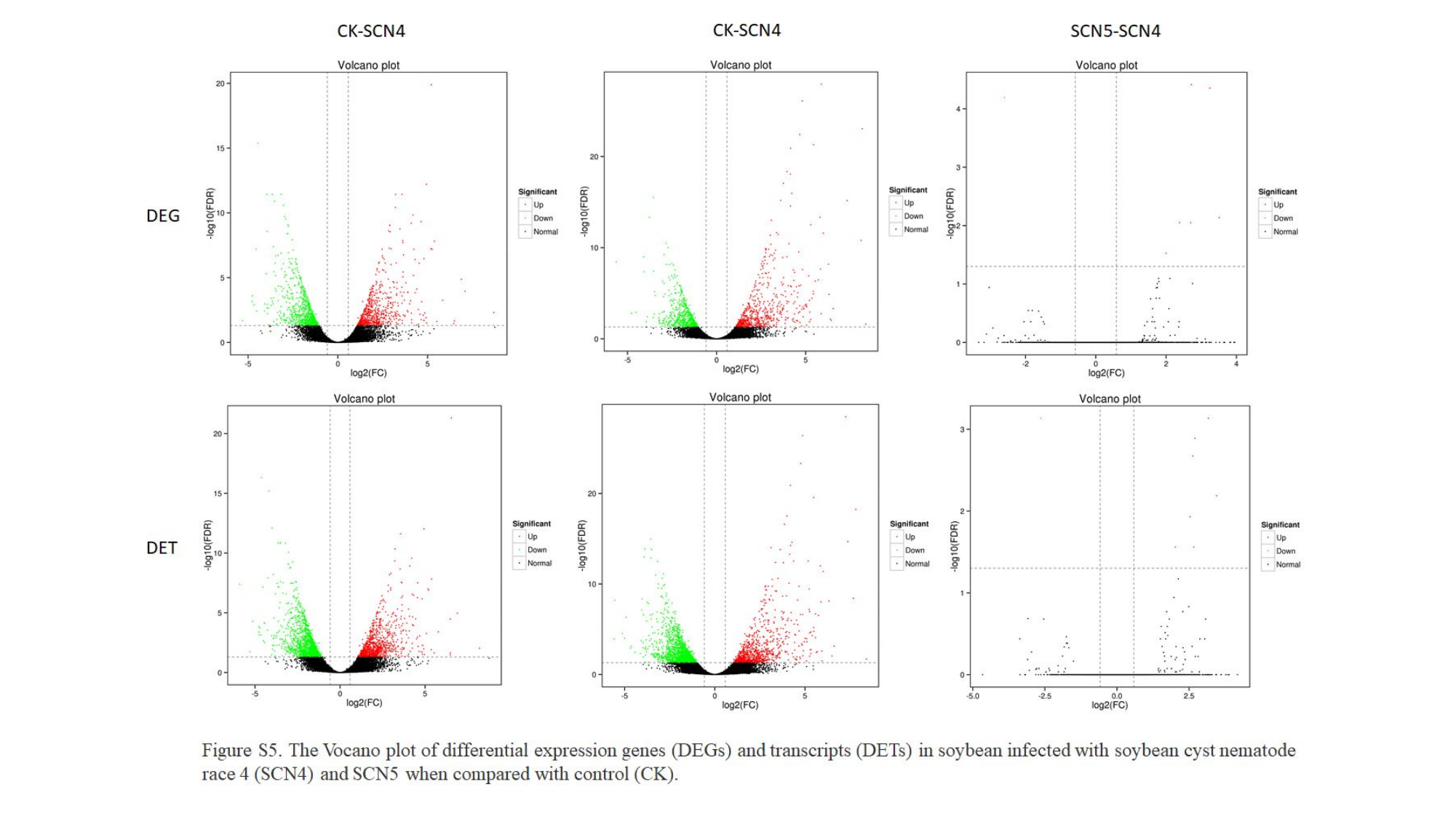

## Slide 6
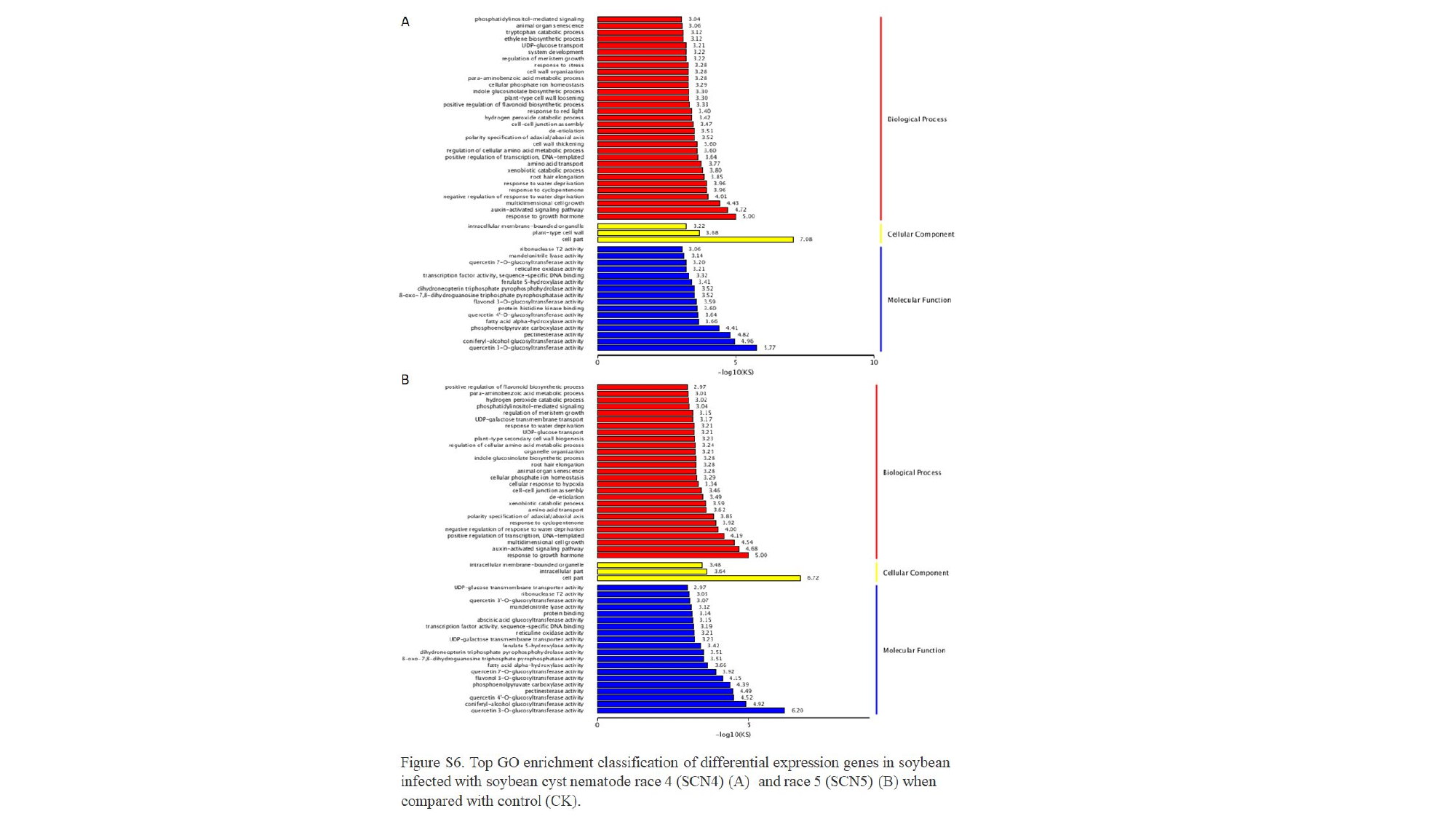

## Slide 7
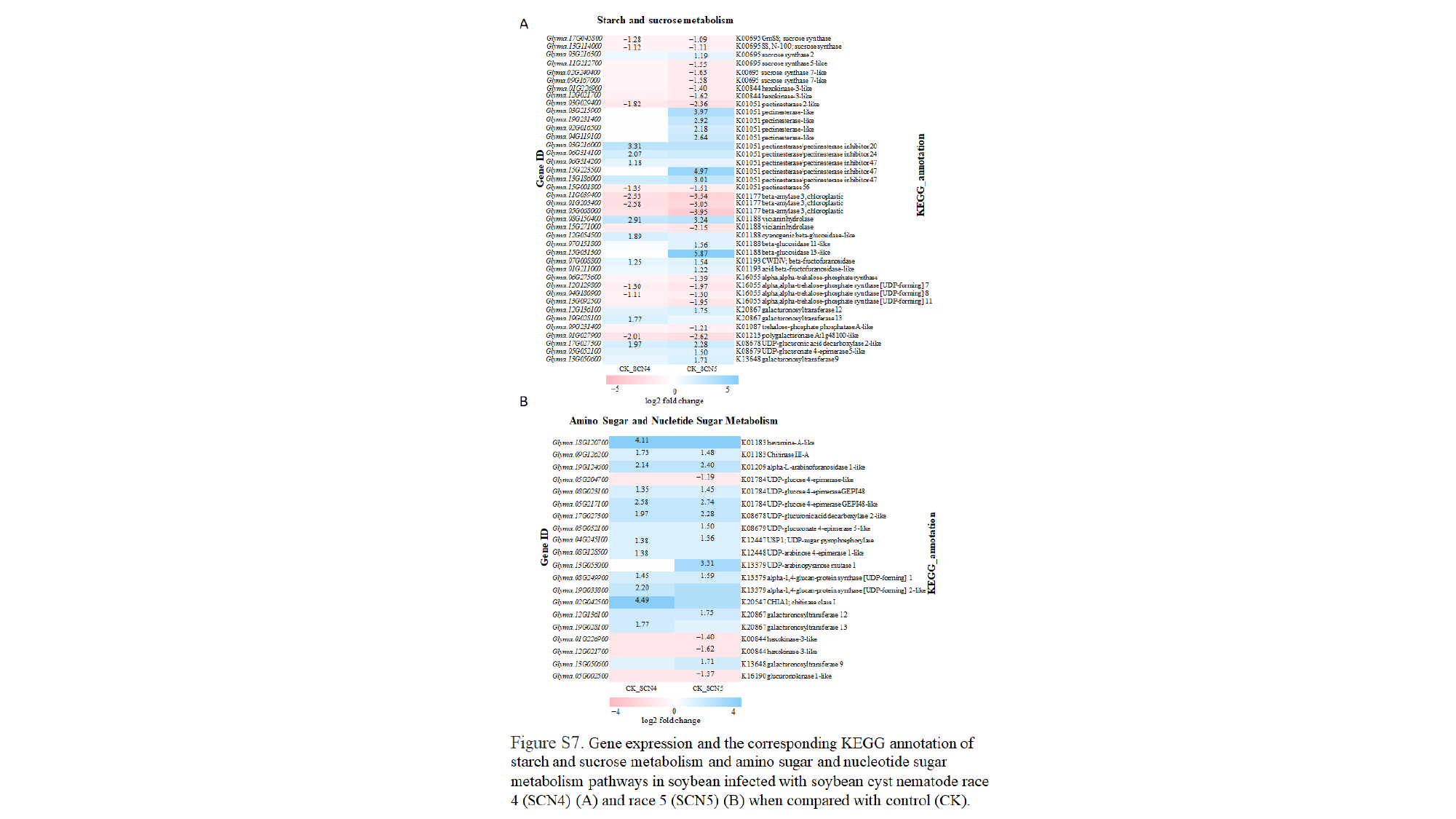

Supplement: Supplementary file 2 [file Presentation_1.PPTX]
